# FOXL2 and NR5A1 induce human fibroblasts into steroidogenic ovarian granulosa‐like cells

**DOI:** 10.1111/cpr.13589

**Published:** 2024-01-08

**Authors:** Fan Wen, Yuxi Ding, Mingming Wang, Jing Du, Shen Zhang, Kehkooi Kee

**Affiliations:** ^1^ The State Key Laboratory for Complex, Severe, and Rare Diseases; SXMU‐Tsinghua Collaborative Innovation Center for Frontier Medicine; Department of Basic Medical Sciences, School of Medicine Tsinghua University Beijing China; ^2^ Reproductive Medicine Center, The First Affiliated Hospital Wenzhou Medical University Wenzhou China; ^3^ Reproductive Medicine Center, Department of Obstetrics and Gynecology, The Second Affiliated Hospital Chongqing Medical University Chongqing China

## Abstract

Human granulosa cells in different stages are essential for maintaining normal ovarian function, and granulosa cell defect is the main cause of ovarian dysfunction. To address this problem, it is necessary to induce functional granulosa cells at different stages in vitro. In this study, we established a reprogramming method to induce early‐ and late‐stage granulosa cells with different steroidogenic abilities. We used an AMH‐fluorescence‐reporter system to screen candidate factors for cellular reprogramming and generated human induced granulosa‐like cells (hiGC) by overexpressing FOXL2 and NR5A1. AMH‐EGFP^+^ hiGC resembled human cumulus cells in transcriptome profiling and secreted high levels of oestrogen and progesterone, similar to late‐stage granulosa cells at antral or preovulatory stage. Moreover, we identified CD55 as a cell surface marker that can be used to isolate early‐stage granulosa cells. CD55^+^ AMH‐EGFP^‐^ hiGC secreted high levels of oestrogen but low levels of progesterone, and their transcriptome profiles were more similar to early‐stage granulosa cells. More importantly, CD55^+^ hiGC transplantation alleviated polycystic ovary syndrome (PCOS) in a mouse model. Therefore, hiGC provides a cellular model to study the developmental program of human granulosa cells and has potential to treat PCOS.

## Graphical abstract

Overexpression of FOXL2 and NR5A1 reprogrammed human fibroblast cells into late‐stage and early‐stage granulosa cells respectively. AMH‐EGFP^+^ hiGCs secret high levels of estradiol and progesterone, and CD55^+^ hiGCs secret high level of estradiol. CD55^+^ hiGC transplantation can alleviate DHEA‐induced PCOS and has potential for cell therapy of PCOS.

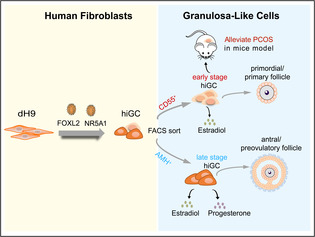

## 1 INTRODUCTION

Granulosa cells are somatic cells that communicate directly with oocytes in follicles and play a crucial role in the development and maturation of oocytes. During mammalian folliculogenesis, developing oocytes are coordinating with different stages of granulosa cells to achieve final oocyte maturations.^1^, ^2^ The primordial follicle consists of a meiosis‐arrested oocyte and a layer of squamous granulosa cells. When these cells become cuboidal and begin to proliferate, the primordial follicle transitions into a primary follicle. A secondary follicle forms when multiple layers of granulosa cells develop around the oocyte, which enlarges but remains meiosis‐arrested. In an antral follicle, the follicular fluid divides granulosa cells into two groups: cumulus granulosa cells surrounding the oocyte and mural granulosa cells lining the follicle wall. Granulosa cells at different stages communicate with the oocyte through gap junctions and secreted factors, playing an important role in oocyte development and maturation.^1^, ^3^, ^4^

The capability of steroid secretions by granulosa cells is critical for ovary development and the reproductive systems.^5^ Granulosa cells convert precursors androgen to estradiol through aromatization reaction. Preovulatory granulosa cells of late‐stage have an increased ability to synthesize progesterone due to upregulation of the genes required to produce progesterone from cholesterol.^6^, ^7^ Transcriptome analysis of human granulosa cells showed that several genes which encode steroidogenic enzymes, including *CYP11A1*, *CYP19A1*, *HSD17B1*, *HSD3B2* were upregulated in antral follicle stage, with peak expression level in the preovulatory stage.^7^ Deficiencies in granulosa cell function impair oocyte development or maturation, resulting in female reproductive diseases such as premature ovarian insufficiency (POI) or polycystic ovary syndrome (PCOS).^8^, ^9^ PCOS is a common reproductive and endocrine disorder in females and a leading cause of infertility.^10^

Currently, cellular model for studying human granulosa cells at different stages are limited. Studies rely on granulosa tumour cell lines,^11^ providing only limited understanding of human granulosa cell development and properties. Direct reprogramming using cell‐type‐specific transcription factor combinations can induce functional cell types from one lineage to another. This strategy has been applied to generate several cell types both in vivo and in vitro.^12^, ^13^ In the reproductive system, mouse and human Sertoli cells have been directly reprogrammed from fibroblasts.^14^, ^15^ In this study, we report the direct reprogramming of human fibroblasts into early‐ and late‐stage granulosa‐like cells (hiGC) through overexpression of FOXL2 and NR5A1. Further characterization confirmed their differential steroidogenic capability and potential to treat PCOS.

## 2 MATERIALS AND METHODS

### 2.1 Cell lines

The H9 ESCs cell line was cultured on feeder cells of mouse embryonic fibroblasts in Knockout DMEM (Thermo Fisher, USA) with 20% KnockOut Serum Replacement (Thermo Fisher, USA), 25 μg/mL bFGF (Gibco, USA), Penicillin–Streptomycin (Gibco, USA), GlutaMAX (Gibco, USA), NEAA (Gibco, USA). The dH9 fibroblast cell line was cultured in high glucose DMEM (Thermo Fisher, USA) with 10% FBS (Gemini, USA), Penicillin–Streptomycin, Glutamax, NEAA.

### 2.2 AMH‐EGFP reporter bearing dH9 cell line establishment

AMH‐EGFP and AMH‐mcherry reporter plasmid with blasticidin resistance element was constructed and used for lentivirus production as previously described.^15^ One day after H9 hESCs colonies were plated, hESCs culture medium was changed to fibroblast culture medium. After outgrowth of colonies changed to fibroblast morphology, cells were passaged. At passage 3, lentivirus of AMH‐EGFP or AMH‐mcherry reporter was transduced to dH9. AMH‐EGFP reporter bearing dH9 of passage 8 was used for reprogramming.

### 2.3 Overexpression vector construction and lentivirus production

Overexpression vectors carrying EF1α promoter followed by cDNA of candidate genes were constructed using the Gateway system (Invitrogen, USA) as previously described.^15^ p2k7 vector without cDNA following EF1α promoter was set as a control for transduction. Lentivirus of six candidate factors or p2k7 were produced with 293FT cell line transfected with vectors of VsVg, △8.9 and overexpressing vectors by Lipofectamine™ 2000 (Invitrogen).

### 2.4 hiGC reprogramming and FACS sorting

For hiGC reprogramming, dH9 cells harbouring AMH‐EGFP/mcherry reporter were transduced with lentivirus followed by one day recovery. From day 0, G418 was added for selection of virally transduced cells for 5 days. At day 7 or day 11, cells were digested with Tryple Express (Thermo Fisher, USA) and single cell suspensions were prepared for FACS sorting. AMH‐EGFP/mcherry positive cells were sorted on Influx cell sorter (BD, USA). For the sorting of CD55 positive cells, digested cells were stained with CD55 antibody for 30 min on ice and washed three times for FACS sorting.

### 2.5 RNA sequencing and analysis

The human cumulus granulosa cells were collected from patients by cutting the cumulus cell layer from each oocyte during in vitro fertilization and were at preovulatory stage. The control cells are fibroblast cells transducted with p2k7 vector without cDNA following EF1α promoter. The AMH‐EGFP^+^ and CD55^+^ cells were sorted using FACS. Total RNA was extracted with TRIZOL (invitrogen) according to manufactures' instructions. 10 ng RNA was used for reverse transcription and pre‐amplified with Smart2 protocol as previously described.^16^ The final cDNA library for sequencing was prepared with TruePrepTM DNA Library Prep Kit V2 for Illumina® (Vazyme, China) and TruePrepTM Index Kit V2 for Illumina® (Vazyme, China). Quality of final cDNA library was checked on an Agilent high‐sensitivity DNA chip (Agilent, USA) followed by sequencing on Illumina Hiseq platform. ClusterProfiler in R was used for analysis of gene ontology (GO). DESeq2 (1.40.2) in R was used to find differentially expressed genes (DEGs). R package of pheatmap was used for creating heatmaps.

For single‐cell transcriptome analysis involving only published data,^7^, ^17^ the single‐cell transcriptome data were converted to TPM values and directly used for comparison (data exhibited as log_2_(TPM+1)) since both used the SMART‐seq2 library construction strategy.

For single‐cell RNA sequencing of hiGC, unsorted FOXL2‐ and NR5A1‐ overexpressed day‐11 reprogrammed cells were subjected to single‐cell RNA sequencing using the BD platform. The Seurat packages (v4.3.0.1) were used for downstream data analysis. Cells were filtered for nCounts (between 2000 and 6000) and mitochondria gene percentage (<10). UMI counts as well as the converted TPM data from published datasets were normalized using the RelativeCount (RC) parameter with scale factor = 10,000 before standard integration steps were performed. Filtered cells were integrated with in vivo collected granulosa and germ cells from published datasets.^7^, ^17^ The integrated data were scaled with the ScaleData function. AMH^+^ and CD55^+^ cells were defined as those with scale data >1 in *AMH* and *CD55* gene, respectively. CD55^+^ and AMH^+^ cells were then selected to go through further integration with the in vivo granulosa cells. DEGs analysis was performed with DESeq2. The heatmap  was created with TBtools(v2.008).

### 2.6 Quantitative real‐time PCRS5

Total RNA was extracted with TRIZOL. Reverse transcription was performed with cDNA synthesis supermix (TRANSGEN, China). Quantitative real‐time PCR (qRT‐PCR) was performed and analysed under the manual instruction of TransStart Green qPCR SuperMix kit (TRANSGEN), Bio‐Rad® CFX Connect™ instrument (Bio‐Rad, USA) and Biorad‐CFX manager software. Relative expression of genes normalized with GAPDH was calculated with ΔΔCq method.

### 2.7 Immunofluorescence staining

For staining of FOXL2 (Abcam, UK), NR5A1 (Abcam), AMH (Abcam), mcherry (Abcam) and CD55 (Abcam), cells sorted by FACS were processed with cytospin centrifuge (Thermo Scientific) at 300 g for 5 min. For staining of CX43, cells were stained on plate. After cells were fixed with 4% paraformaldehyde and penetrated with 1‰ triton X‐100, cells were blocked with 10% FBS and then incubated overnight at 4°C with primary antibodies. Then cells were incubated with Alexa Fluor 488 or 594 conjugated secondary antibodies (Invitrogen). DAPI was used to stain nuclear. Slides were then mounted with Prolong Diamond Anti‐Fade Mounting Reagent (Thermo Fisher) and cover slip.

### 2.8 Western blotting analysis

Cell lysates were prepared using radioimmune precipitation assay lysis buffer (RIPA) lysis buffer. Protein was electrophoresed under reducing conditions in SDS‐PAGE gels and transferred to nitrocellulose membranes. Membranes were incubated overnight at 4°C with primary antibodies. Then Membranes were incubated with Goat anti‐rabbit antibody or rabbit anti‐goat antibody for 1 h at room temperature.

### 2.9 Steroidogenic assays

The sorted AMH^+^ or CD55^+^ hiGC were plated on 96 wells plates as a density of 6 × 10^4^ cells/well. After 24 h, these cells were treated with 100 ng/mL testosterone, and cultured for 48 h. 5.5 IU/mL FSH or 60 ng/mL Activin A was added simultaneously with testosterone when testing the effect of FSH and Activin A. The culture medium was harvested for measurement of estradiol or progesterone levels. Estradiol levels were measured with Cayman's Estradiol ELISA Kit (Cayman chemical, USA). Progesterone levels were measured with Progesterone ELISA Kit (Sangon Biotech, China). Each experiment was repeated three times.

### 2.10 hGDF9:BMP15 heterodimer purification, treatment of mouse cumulus cells and hiGC

293FT cells were transfected with hGDF9:BMP15 expressing plasmid with VigoFect transfection reagent (Vigorous, China) for protein production. Supernatant was harvested for purification of heterodimer using anti‐FLAG M2 affinity gel (Sigma‐Aldrich, USA) and 3 × FLAG peptide (Sigma‐Aldrich, USA). Mouse cumulus cells were collected and treated with hGDF9:BMP15 as previously described.^3^ hiGC were treated with hGDF9:BMP15 for 48 h before RNA was extracted for qRT‐PCR analysis.

### 2.11 Co‐culture of hiGC or mouse granulosa cells with mouse GV stage oocyte

Female prepuberal (21‐day‐old) mice of the CD‐1 strain were injected with 5 IU pregnant mare's serum gonadotropin and ovarian follicles were collected at 44–48 h post injection. GV stage oocytes were harvested under stereo microscope. hiGC or mouse granulosa cells were plated onto 96 wells plate at 12 h before co‐culture and covered with paraffin oil. GV stage oocytes were plated onto wells plated with hiGC or mouse granulosa cells or control wells. GVBD and first body extrusion were observed with live imaging (Nikon, Ti2‐E, Japan).

### 2.12 Establishment of PCOS model and hiGC transplantation

C57BL/6J mice were purchased from Vital River Laboratory Animal Technology Co., Ltd (Beijing, China). PCOS model establishment was modified from previous study.^18^ In brief, 21‐day‐old female mice of the C57BL/6 strain received daily subcutaneous injection of DHEA (HY‐14650; MCE; 18 mg per 100 g body weight, dissolved in sesame oil) for 30 days. DHEA+OC group were provided with a specially customized diet containing 2 mg/kg ethinylestradiol and 200 mg/kg norgestrel with free access^19^ from day 31 to mimic the clinical treatment of PCOS. DHEA+hiGC group received bilateral kidney capsule transplantation of CD55^+^ hiGC, with 1.5 × 10^6^ cells each side. DHEA and DHEA+OC group received sham operation. Subcutaneous injection of DHEA was continued for 8 days and all mice were sacrificed at 15 days after transplantation.

### 2.13 GTT analysis

GTT analysis was conducted as previously descried.^18^ Mice were fasted for 12 h before experiment. Tail vein blood samples were collected for measurement of glucose levels. After measurement of fasting glucose level, mice were intraperitoneally injected with D‐glucose (2 g/kg bodyweight). Glucose levels were measured at 15, 30, 60, 90, 120 min after D‐glucose injection.

### 2.14 Histological analysis

Ovaries were fixed in 4% paraformaldehyde overnight and then embedded in paraffin. Sections of 5 μm thickness were prepared and stained with haematoxylin and eosin. Four sections were randomly selected from the middle of each ovary for analysis. Images were taken by a light microscope. The numbers of corpus lutea and cystic follicles were counted by an investigator blind to the treatment group.

### 2.15 Immunohistochemistry

Immunohistochemistry was performed using Solarbio SP Kit (Broad Spectrum) (SP0041) following manufacturers' guidance. Briefly, paraffin sections were rehydrated, retrieved of antigen (using citrate buffer pH = 6.0), depleted of endogenous catalase, blocked under room temperature, and sequentially incubated with primary and secondary antibodies. The samples were then incubated with Streptavidin‐POD and signal were visualized by adding DAB solution. The primary antibody used in this study was rabbit anti‐CD55 (ER1802‐46).

### 2.16 Quantification and statistical analysis

All data from at least three independent measurements were presented as means ± SEM. Student's *t*‐tests and one‐way ANOVA were used where appropriate for statistical analyses in this research with prism 8.0 software. A *p* value <0.05 was considered as statistically significant. *p < 0.05, **p < 0.01 and ***p < 0.001.

## 3 RESULTS

### 3.1 FOXL2 and NR5A1 combination induce AMH‐EGFP ^+^ cells

**FIGURE 1:**
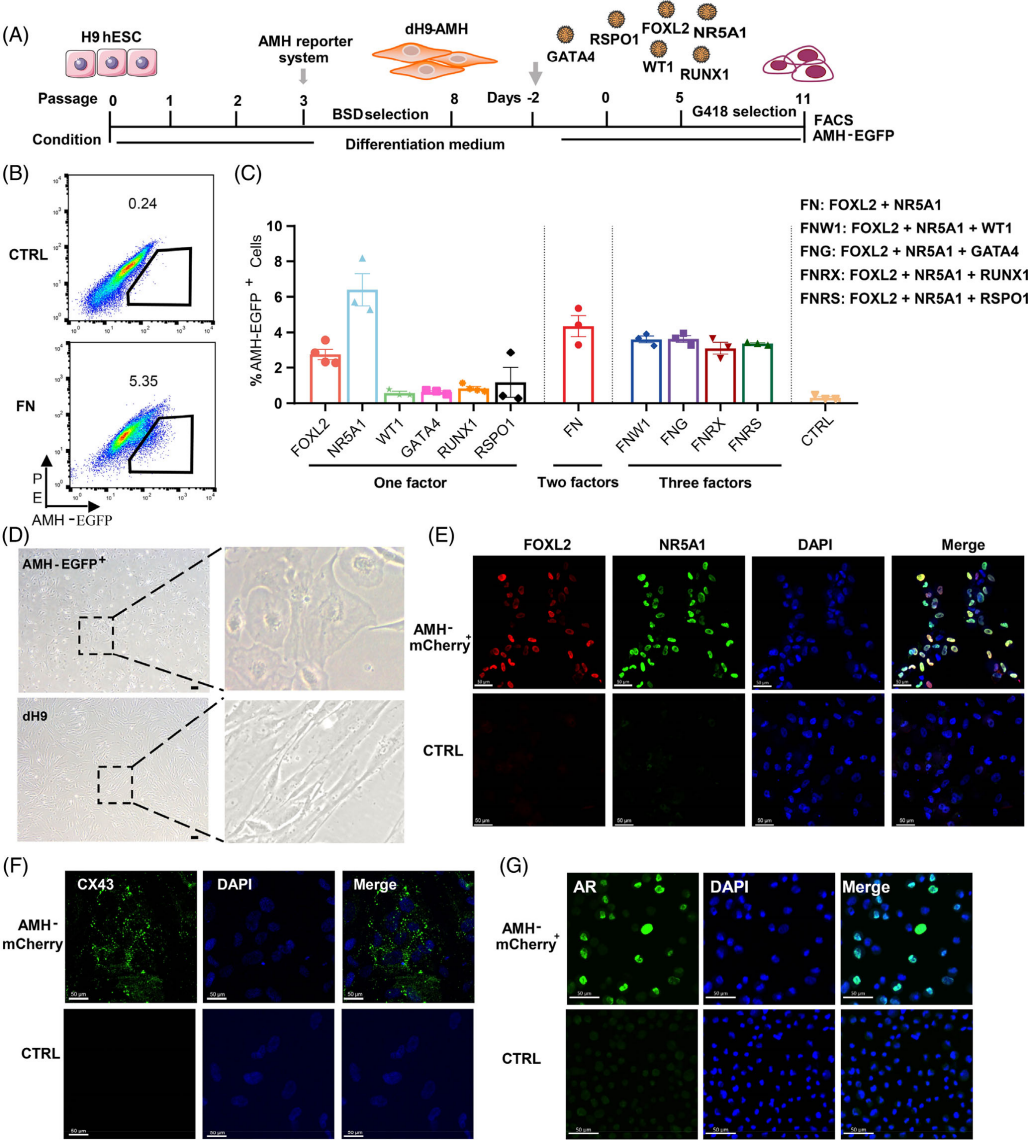
FOXL2 and NR5A1 induce AMH‐EGFP ^+^ cells. (A) Scheme of the establishment of dH9‐AMH cell line and the time line of reprogramming. (B) Percentages of AMH‐EGFP^+^ cells were analysed by flow cytometric analysis (FACS) on day 11 post G418 selection. CTRL group was dH9 transduced with the control empty lentivirus. (C) Statistical analysis of %AMH‐EGFP^+^ cells in different groups. *n* = 3 or 4, and data were presented as mean ± SEM.(D) The morphology of AMH‐EGFP^+^ cells sorted from FN reprogramming system. (E) Immunofluorescence staining of FOXL2 and NR5A1 in day 11 sorted AMH‐mCherry^+^ cells. Scale bar = 200 μm. (F) Immunofluorescence staining of CX43 in day 11 sorted AMH‐mCherry^+^ cells. (G) Immunofluorescence staining of AR in day 11 sorted AMH‐mCherry^+^ cells.

To isolate granulosa‐like cells from heterogeneous reprogrammed cells, we constructed gene reporter systems carrying the AMH promoter regulating the expression of fluorescent proteins EGFP or mCherry (Figure S1). We compared single‐cell RNA sequencing data of human fetal^17^ and adult ovarian granulosa cells,^7^ confirming that AMH is expressed in adult but not fetal granulosa cells (Figure S1). To avoid the potential tumorigenicity of cells directly derived from pluripotent embryonic stem cells and get abundant cells for future experiments, human fibroblasts (dH9) were derived from the human embryonic stem cell (hESC) line H9 as previously described.^15^ Differentiated dH9 cells exhibited fibroblast morphology distinct from undifferentiated hESCs (Figure S1). More importantly, expression levels of pluripotent genes were diminished and fibroblast‐related genes were highly expressed in dH9 cells compared to hESCs (Figure S1). Hence, we established a dH9‐AMH cell line bearing AMH fluorescence reporter systems (Figure 1A).

Based on previous reports on granulosa cell developmental programs,^20^, ^21^, ^22^, ^23^, ^24^, ^25^ six key factors (FOXL2, NR5A1, WT1, GATA4, RUNX1, RSPO1) are important regulators of mouse granulosa cells. We analysed the single‐cell RNA sequencing data of human gonadal^17^ and adult ovarian granulosa cells,^7^ confirming the expression of these factors in human granulosa cells (Figure S1). Thereafter, we selected homologues of these six genes in humans as candidates for reprogramming human fibroblasts into granulosa cells.

To obtain the minimal induction factor, we tested the reprogramming percentage of single factors and added more factors to test if they increased the percentage of putative granulosa cells. The AMH‐EGFP^+^ percentage was measured by FACS at day 11 after lentiviral transduction (Figure 1A). Results showed that FOXL2 or NR5A1 single factor induction yielded 2.8% and 6.4% AMH‐EGFP^+^ cells on average respectively, higher than other single factors and the p2k7 empty vector lentiviral control (Figure 1B,C). Hence, we selected FOXL2 and NR5A1 to test combined induction efficiency. The induction efficiency of AMH‐EGFP by FOXL2 and NR5A1 combination (FN) was 4.4% on average, higher than that of FOXL2 alone but lower than that of NR5A1 alone. Adding other single factors to the FN combination did not further increase AMH‐EGFP induction efficiency. Thus, NR5A1 and FN combination were selected for further analysis.

NR5A1 also expresses in testicular Sertoli cells and has been used for in vitro reprogramming of fibroblast cells into Sertoli cells.^14^, ^15^, ^23^ However, FOXL2 exclusively expresses in female gonads and plays a key role in regulating granulosa cell development programs.^21^, ^24^, ^26^ We found that male gonadal gene *SOX9* expression was higher in NR5A1 cells but female gonadal gene *FST* expression was significantly higher in the FN group. Moreover, granulosa‐related gene *AR* expression was significantly higher in the FN group compared to the NR5A1 group (Figure S1). Thus, single NR5A1 factor may not be sufficient for specifying granulosa cell identity, but FN combination induced AMH‐EGFP^+^ cells are more likely to be granulosa‐like cells.

The AMH‐EGFP^+^ cells induced by FN combination showed epithelial‐like morphology on day 11, different from dH9 fibroblast morphology (Figure 1D). To validate the reliability of our fluorescent reporter system and facilitates the use of different fluorescent antibodies in various conditions, we confirmed that FN could also reprogram human fibroblasts carrying AMH‐mCherry reporter systems. The induction efficiency of AMH‐mCherry reporter system was about 5.0%, comparable to that of AMH‐EGFP reporter system (Figure S2A and Figure 1B). Protein expression of AMH and mCherry was confirmed in sorted AMH‐mCherry^+^ cells (Figure S2C). Overexpression of FOXL2 and NR5A1 was confirmed by immunofluorescence (Figure 1E) and western blot analysis (Figure S2C). Gap junction protein CX43 and AR were detected in AMH‐mCherry^+^ cells (Figure 1F,G).

We further tested the FN reprogramming system on primary human fetal lung fibrolast cells (WI‐38). During continuous culture, a portion of the WI‐38 cells transfected with FN showed substantial morphology change (Figure S3A). This transformation was similar to what was observed in FN‐overexpressed dH9 cells. We also detected the induction of AMH‐EGFP^+^ and CD55^+^ cells at day 4, 7, and 11 of culture (Figure S3B,C). The results showed that CD55^+^ cells were detectable from day 4 and were present until day 11 (Figure S3B). The positive rate of CD55 reached its highest of 39.98% at day 7 of culture. AMH‐EGFP^+^ cells were also detectable from day 4 of culture, with a relatively lower positive rate of ~5% (Figure S3C). The above results suggested that the FN reprogramming system is repeatable in primary human fibroblast cells and added to the evidence that granulosa‐like cells could be successfully derived from human fibroblasts.

### 3.2 AMH‐EGFP ^+^ cells have a similar transcription profile to cumulus granulosa cells

**FIGURE 2:**
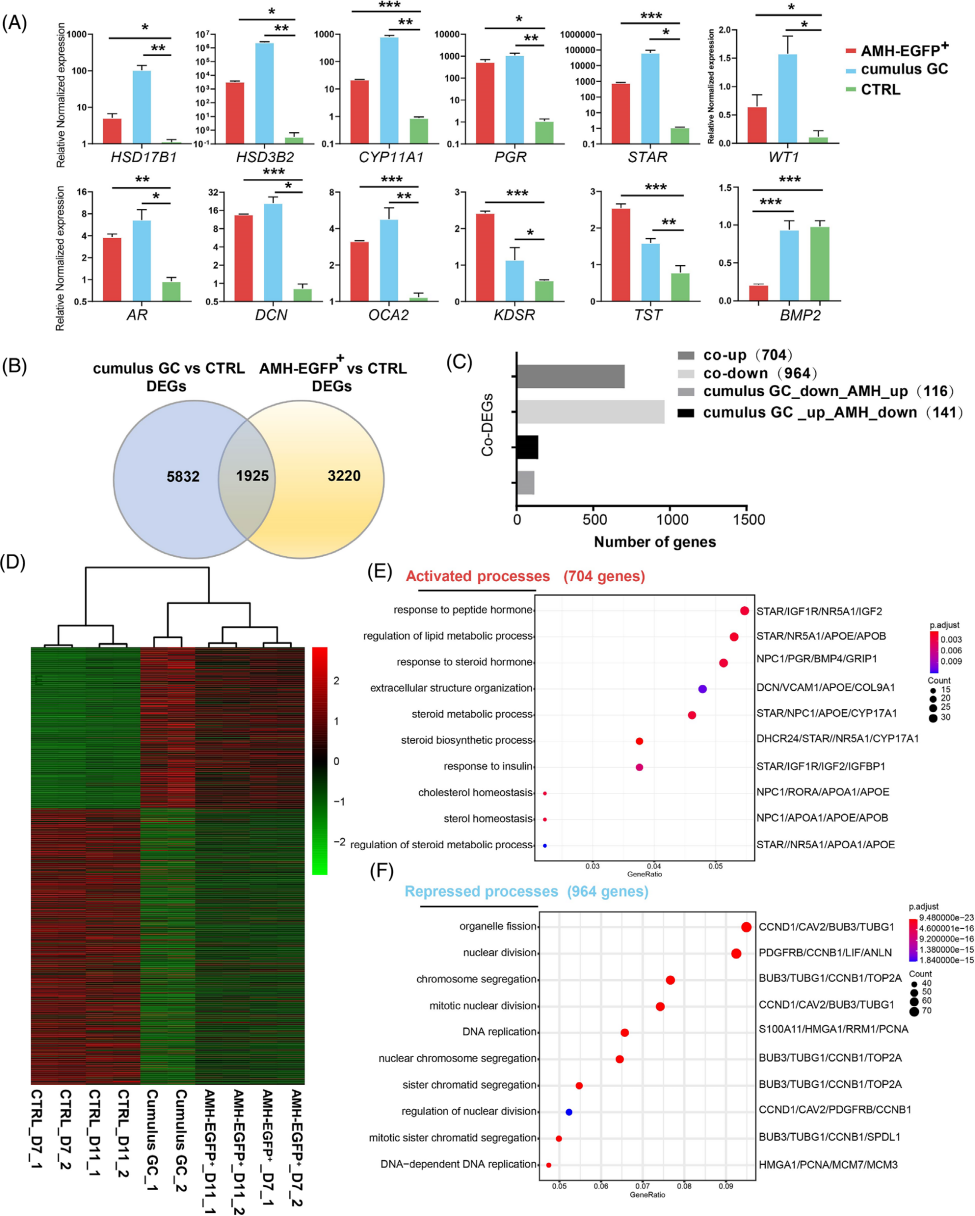
AMH‐EGFP ^+^ cells resemble human cumulus granulosa cells in transcriptome analysis. (A) The expression of granulosa related genes in day 11 AMH‐EGFP^+^ cells, human cumulus granulosa cells (cumulus GC) and CTRL group cells were measured by qRT‐PCR. CTRL group was dH9 transduced with the control empty lentivirus. Expression were normalized to *GAPDH. n* = 3 and data were presented as mean ± SEM. *p* values were determined by one‐way ANOVA. *p < 0.05, **p < 0.01 and ***p < 0.001. (B) Venn plot showing the DEGs between day 7 AMH‐EGFP^+^ cells and CTRL group, DEGs between cumulus GC and CTRL group, and Co‐DEGs of the two sets. (C) Bar chart showing the numbers of different types of Co‐DEGs. (D) Heatmap of transcriptome profile of day 7 and day 11 AMH‐EGFP^+^ cells, cumulus GC and CTRL group cells of day 7 and day 11. (E) GO analysis of genes co‐upregulated in hiGC and cumulus GC. (F) GO analysis of genes co‐downregulated in hiGC and cumulus GC.

qRT‐PCR analysis showed that the expression levels of granulosa gene markers *HSD17B1*, *HSD3B2*, *CYP11A1*, *PGR*, *STAR*, *AR*, *WT1*, *BMP2* were significantly upregulated in AMH‐EGFP^+^ cells and human cumulus granulosa cells (cumulus GC, clinical samples collected by cutting the cumulus cell layer from each oocyte during in vitro fertilization and were at preovulatory stage) compared to the control cells (fibroblast cells transducted with p2k7 vector without cDNA following EF1α promoter). The expression levels of genes identified in single‐cell analysis of granulosa cells were also significantly upregulated in AMH‐EGFP^+^ cells and cumulus GC (Figure 2A).

Transcription profiles of day 7 and day 11 AMH‐EGFP^+^ cells were compared with human cumulus cells and control cells by RNA sequencing. There were 7757 differentially expressed genes (DEGs) between cumulus GC and the controls, 5145 DEGs between AMH‐EGFP^+^ cells and the controls, and 1925 common DEGs (Co‐DEGs) between the cumulus GC and AMH‐EGFP^+^ cells (Figure 2B). Among the 1925 Co‐DEGs, 704 genes were co‐upregulated and 964 genes were co‐downregulated (Figure 2C), indicating that most of the Co‐DEGs had the same transcriptional trends in AMH‐EGFP^+^ cells and cumulus GC. Hierarchical clustering analysis with Co‐DEGs showed that, AMH‐EGFP^+^ cells and cumulus GC had a similar expression pattern, and the AMH‐EGFP^+^ cells sorted on day 7 and day 11 were similar to each other (Figure 2D).

We further analysed the biological process related to Co‐DEGs with gene ontology analysis. 704 co‐upregulated genes were enriched in GO terms important to granulosa functions such as ‘steroid biosynthetic process’, ‘response to steroid hormone’ and ‘cell‐cell junction organization’ (Figure 2E). On the other hand, the 964 co‐downregulated genes were enriched in GO terms related to cellular proliferation such as ‘nuclear division’, ‘chromosome segregation’ and ‘DNA replication’ (Figure 2F). The GO analysis suggested that steroidogenesis was activated and cellular proliferation was downregulated in AMH‐EGFP^+^ cells as in cumulus granulosa cells. Taken together, the above results showed that FN combination induced AMH‐EGFP^+^ cells acquired transcriptome profile similar to human cumulus granulosa cells.

### 3.3 AMH‐EGFP ^+^ cells secreted high levels of estradiol and progesterone

**FIGURE 3:**
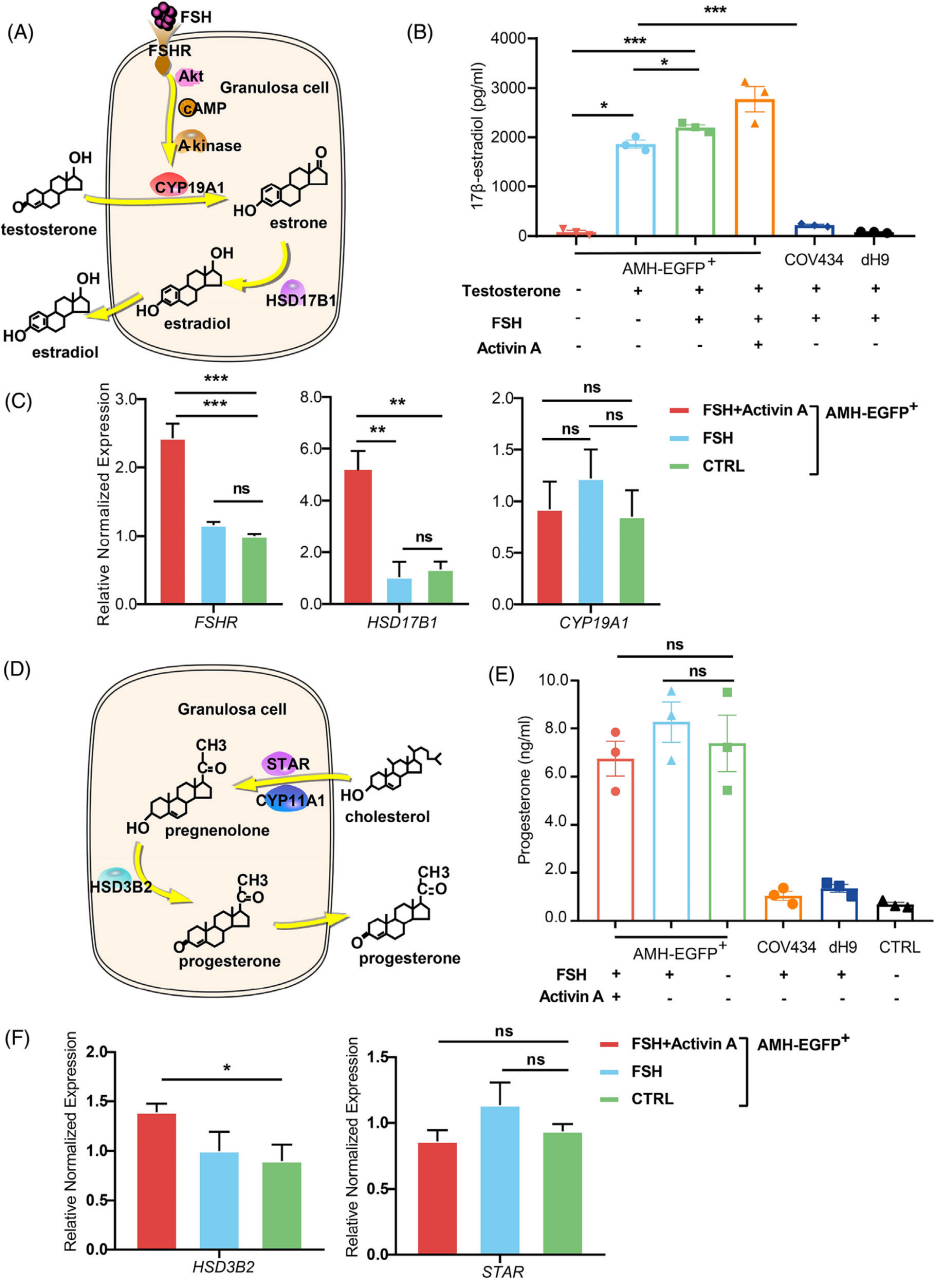
AMH‐EGFP ^+^ cells possess steroidogenic activity of estradiol and progesterone. (A) Scheme of estradiol synthesis pathway in granulosa cells. (B) ELISA measurement of 17β‐estradiol level in culture medium collected at 48 h after treatment. (*n* = 3). (C) The expression of *CYP19A1, HSD17B1, FSHR* in FSH and Activin A treated AMH‐EGFP^+^ cells were measured by qRT‐PCR (*n* = 3). Expression were normalized to *GAPDH*. (D) Scheme of progesterone synthesis pathway in granulosa cells. (E) ELISA measurement of progesterone level in culture medium collected at 48 h after treatment (*n* = 3). CTRL was culture medium. (F) The expression of *HSD3B2* and *STAR* in FSH and Activin A treated AMH‐EGFP^+^ cells were measured by qRT‐PCR (*n* = 3). Expression were normalized to *GAPDH*. All data were presented as mean ± SEM. p values were determined by one‐way ANOVA. *p < 0.05, **p < 0.01 and ***p < 0.001; ns, no statistical significance.

Estradiol is one of the key steroid hormones synthesized by granulosa cells. Aromatase CYP19A1 catalyses the conversion of androgen such as testosterone to estrone which is further converted to estradiol by HSD17B1^27^ (Figure 3A). High level of 17β‐estradiol (1739.2–2014.1 pg/mL) was detected in the culture media of AMH‐EGFP^+^ cells in the presence of testosterone, but not in the media without testosterone (Figure 3B). In addition, we found that the estradiol level secreted by AMH‐EGFP^+^ cells was enhanced in response to FSH stimulation, and the combined treatment of Activin A and FSH further stimulated the production of estradiol (Figure 3B). FSH stimulation alone slightly upregulated the expression of *FSHR* and *CYP19A1,* while combined treatment with Activin A and FSH significantly upregulated the expression of *HSD17B1* and *FSHR* but not *CYP19A1* (Figure 3C). However, the estradiol level of a granulosa cell line, COV434, was 8.5 folds lower than AMH‐EGFP^+^ cells with the addition of FSH. These results indicated that AMH‐EGFP^+^ cells can convert testosterone to estradiol and this catalytic activity is enhanced by FSH and activin A.

Another steroid hormone produced by granulosa cells is progesterone. Cholesterol transported into granulosa cells is initially catalysed to pregnenolone by CYP11A1, followed by conversion of pregnenolone to progesterone by HSD3B2^27^ (Figure 3D). AMH‐EGFP^+^ cells secreted significantly higher level of progesterone (5.4–9.5 ng/mL) than control or COV434 cells (Figure 3E). The addition of FSH or Activin A did not increase progesterone level of AMH‐EGFP^+^ cells (Figure 3E). In these cells, the expression of *HSD3B2* was upregulated by FSH and Activin A, but the expression of *STAR* was not affected (Figure 3F).

### 3.4 Identification of CD55 as a granulosa cell surface marker expressed in hiGC

**FIGURE 4:**
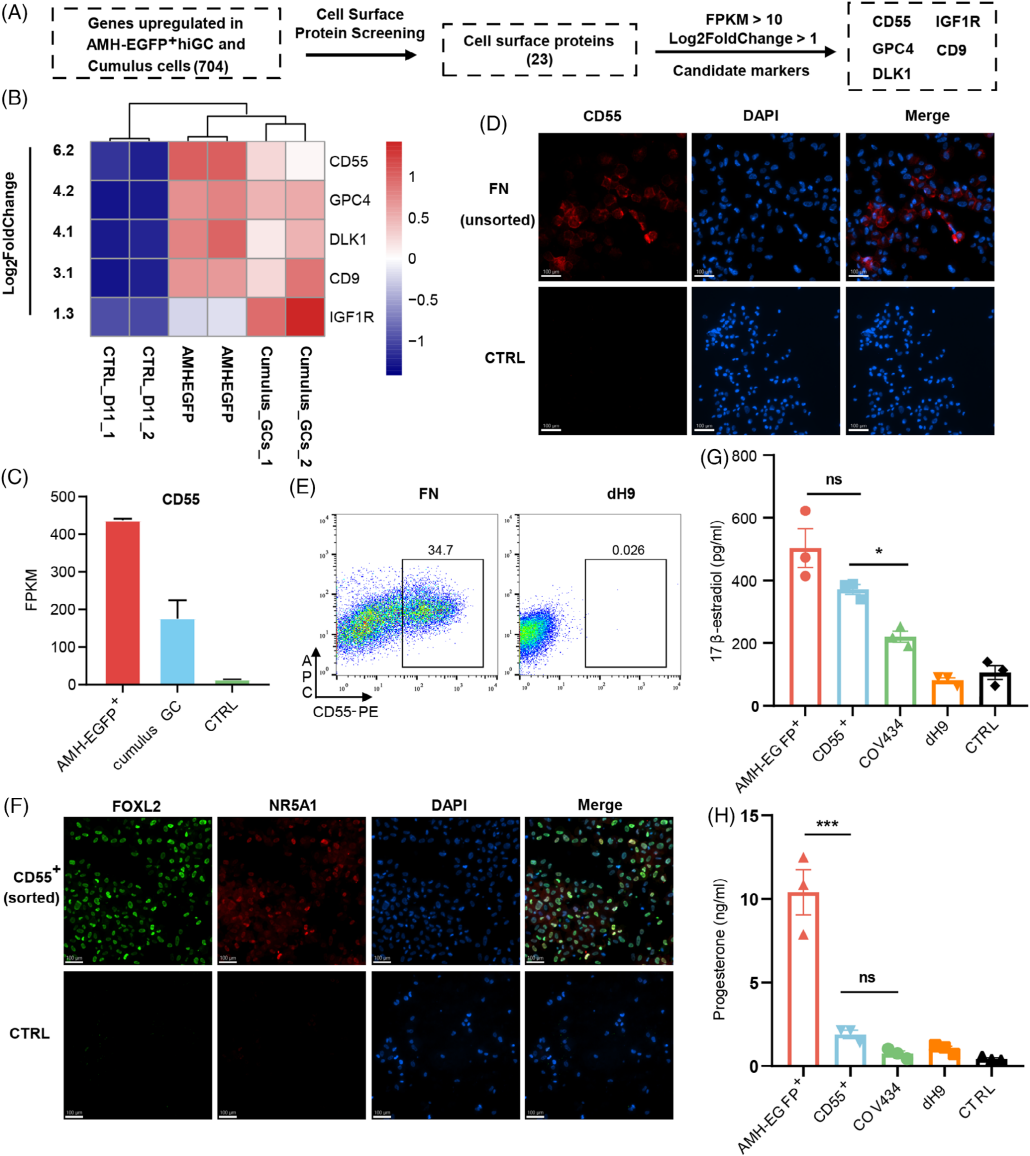
Identify CD55 as a granulosa surface marker expressed in hiGC. (A) Scheme of surface marker screening criteria. (B) Heatmap showing the Log_2_FoldChange of 5 surface markers screened from 704 genes. CTRL group was transduced with p2k7 empty virus. (C) FPKM of CD55 in AMH‐EGFP^+^ hiGC, cumulus GC and CTRL group. (D) Immunofluorescence staining of CD55 in unsorted cells of FN group and dH9 group. Scale bar = 100 μm. (E) Flow cytometric analysis of CD55^+^ cells in FN group and dH9 group. (F) Immunofluorescence staining of FOXL2 and NR5A1 in day 11 sorted CD55^+^ cells. Scale bar = 100 μm. (G) ELISA measurement of 17β‐estradiol level in culture medium collected at 48 h after treatment (*n* = 3). CTRL was culture medium. (H) ELISA measurement of progesterone level in culture medium collected at 48 h after treatment (*n* = 3). CTRL was culture medium. All data were presented as mean ± SEM. p values were determined by one‐way ANOVA. *p < 0.05, and ***p < 0.001; ns, no statistical significance.

To identify a cell surface marker for hiGC sorting, we screened genes enriched in AMH‐EGFP^+^ hiGC and cumulus cells (Figure 4A). Among the 704 co‐upregulated DEGs, five genes (*CD55*, *GPC4*, *DLK1*, *CD9*, and *IGF1R*) of 23 cell‐surface‐expressing markers met the following criteria: (1) FPKM >10; (2) Log_2_ FoldChange >1. 5 (Figure 4B). CD55 showed the highest fold change of enrichment and was expressed at low levels in controls (Figure 4C), so it was selected for further analysis. Analysis of single‐cell RNA sequencing data of human fetal and adult granulosa cells showed that *CD55* is abundantly expressed from 14 weeks onward^7^, ^17^ (Figure S4A). Immunohistochemistry staining of CD55 in human ovarian section showed that, positive CD55 signal was observed in all follicle developmental stages. In late antral follicles, cumulus granulosa cells showed stronger CD55 expression than mural cells (Figure S4B). Immunostaining analysis confirmed CD55 expression on many unsorted cells after FN overexpression but not in control fibroblasts (Figure 4D). Flow cytometric analysis showed that about 34.7% of FN induced cells belonged to a strong CD55^+^ population (Figure 4E). Moreover, almost all sorted CD55^+^ cells showed high expression of FOXL2 and NR5A1 (Figure 4F).

Next, CD55^+^ cells were tested for their steroidogenic properties. CD55^+^ cells secreted high level of 17β‐estradiol (about 371.3 pg/mL), significantly higher than COV434 and the control, but slightly lower than AMH‐EGFP^+^ cells (Figure 4G). Notably, CD55^+^ cells secreted significantly lower level of progesterone than AMH‐EGFP^+^ hiGC, and at similar level with COV434 (Figure 4H). Progesterone is abundantly produced by late‐stage granulosa cells, the secretion of high levels of estradiol but low levels of progesterone by CD55^+^ cells inferred that they might be early‐stage granulosa cells.

### 3.5 CD55 ^+^ AMH‐EGFP ^−^ cells have similar properties as early‐stage granulosa cells

**FIGURE 5:**
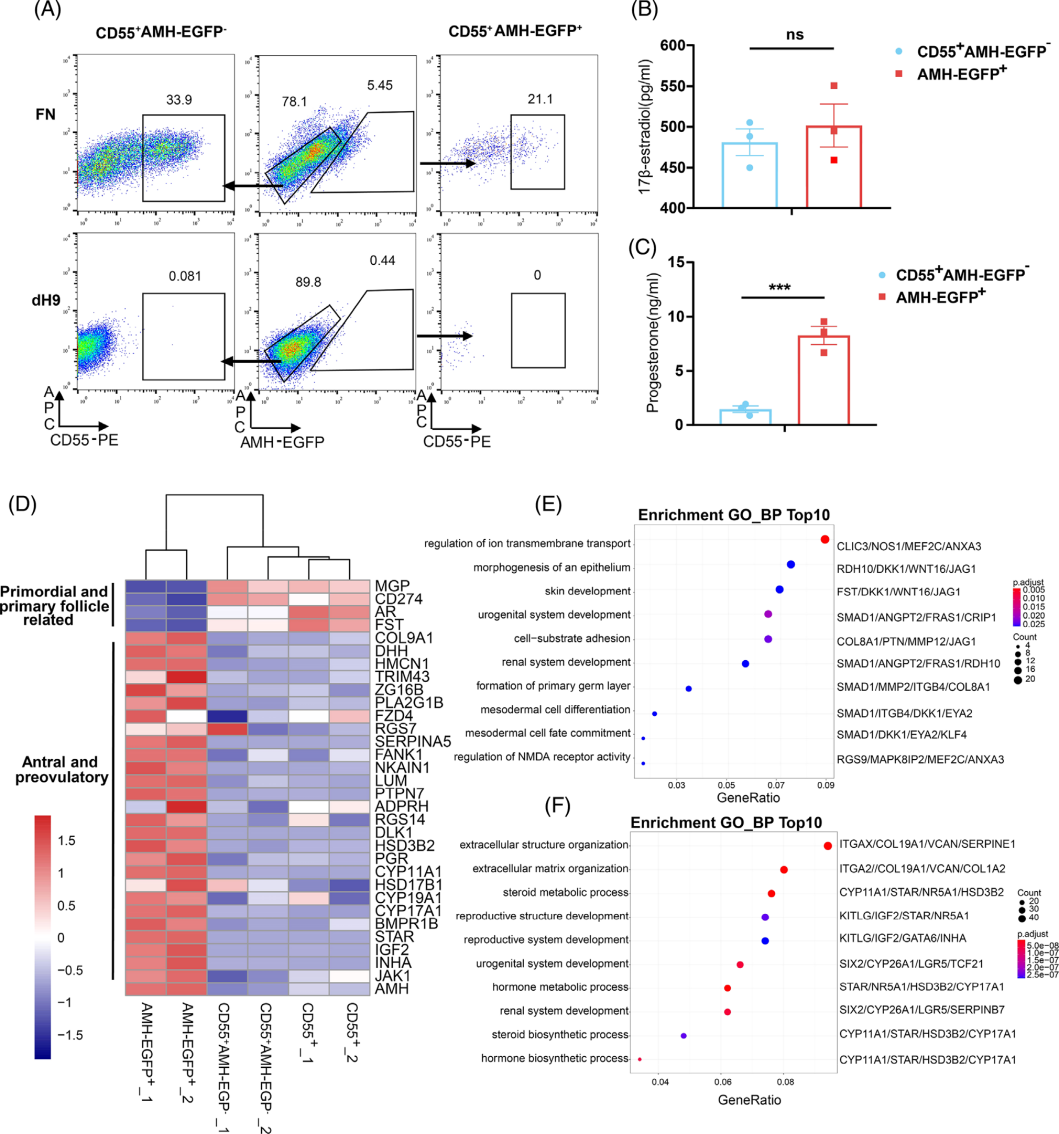
CD55 + AMH‐EGFP‐ cells are early‐stage hiGC. (A) Flow cytometric analysis of CD55^+^ and AMH‐EGFP^+^ cells in FN and dH9 group. (B) ELISA measurement of 17β‐estradiol level in culture medium collected at 48 h after treatment (*n* = 3). (C) ELISA measurement of progesterone level in culture medium collected at 48 h after treatment (*n* = 3). (D) Heatmap showing the expression of early‐ and late‐stage granulosa cells related genes in AMH‐EGFP^+^, CD55^+^AMH‐EGFP^−^ and CD55^+^ cells. (E) GO analysis of genes upregulated in CD55^+^AMH‐EGFP^−^ cells compared to AMH‐EGFP^+^ cells. Top10 GO term of biological process were showed. (F) GO analysis of genes upregulated in AMH‐EGFP^+^ cells compared to CD55^+^AMH‐EGFP^−^ cells. Top10 GO term of biological process were showed. All data were presented as mean ± SEM. *p* values were determined by one‐way ANOVA. ***p < 0.001; ns, no statistical significance.

To test whether CD55^+^ cells correspond to an early population, we analysed the percentage of CD55^+^ cells in AMH‐EGFP^−^ and AMH‐EGFP^+^ cells. Among the 78.1% AMH‐EGFP^−^ cells, there were approximately 33.9% CD55^+^ cells in contrast to only 21.1% CD55^+^ cells in the 5.45% AMH‐EGFP^+^ populations (Figure 5A). Hence, FN induced approximately 26% CD55^+^ AMH‐EGFP^−^ cells, but only approximately 1.1% CD55^+^AMH‐EGFP^+^ cells. CD55^−^ AMH‐EGFP^+^ cells accounted for 78.9% of AMH‐EGFP^+^ cells and was assumed to be the main cell type that contributes to properties of AMH‐EGFP^+^ cells. Steroidogenic tests confirmed that CD55^+^AMH‐EGFP^−^ cells secreted high level of 17β‐estradiol which was comparable to AMH‐EGFP^+^ cells but much lower of progesterone than AMH‐EGFP^+^ cells (Figure 5B,C).

Analysis of transcriptomes of CD55^+^, CD55^+^AMH‐EGFP^−^ cells, and AMH‐EGFP^+^ cells indicates that the expression levels of primordial‐ or primary‐follicle granulosa‐cells marker genes^7^, ^28^, ^29^, ^30^ were higher in CD55^+^ and CD55^+^AMH‐EGFP^−^ cells. Especially, early granulosa marker *FST* was significantly higher in CD55^+^ cells than in AMH‐EGFP^+^ cells (Figure S4D), and the existence of FST protein in CD55^+^ cells was verified using immunostaining (Figure S4C). In addition, gene markers enriched in antral or preovulatory genes were higher in AMH‐EGFP^+^ cells (Figure 5D). GO terms of DEGs upregulated in CD55^+^AMH‐EGFP^−^ included biological process of ‘morphogenesis of an epithelium’, ‘urogenital system development’, and ‘mesodermal cell differentiation’ (Figure 5E), suggesting the mesonephros and epithelium origination of primordial follicle granulosa cells. In contrast, GO terms of DEGs upregulated in AMH‐EGFP^+^ hiGC included ‘extracellular matrix organization’, ‘steroid metabolic process’, ‘steroid biosynthetic process’ (Figure 5F), suggesting that these hiGC had the properties of higher steroid secretion and extracellular matrix production as in late‐stage granulosa cells.

We further performed single‐cell sequencing of unsorted, day‐11 reprogrammed fibroblasts. Filtered cells were integrated with in vivo collected granulosa cells from published datasets.^7^, ^8^, ^17^ CD55^+^ and AMH‐EGFP^+^ cells were then selected to go through further integration with the in vivo granulosa cells. As shown in Figure S5A, the majority of CD55^+^ cells fall in between late adult granulosa (antral and preovulatory, upper left part) and fetal granulosa cells (lower part), partially overlapping with adult early and fetal granulosa cells. We further analysed the DEGs between AMH‐EGFP^+^ and CD55^+^ hiGC. The results showed that early granulosa cell marker *FST* was enriched in CD55^+^ hiGC, with a positive rate of 69.6%, while several preovulatory granulosa cell markers (*TNC*, *SNAI2*, *GDF15*, *COL6A3*, and *TFPI*)^7^ were enriched in AMH‐EGFP^+^ hiGC, in which the positive rate of *TNC* was 33.3% and *SNAI2* was 36.4% (Figure S5B). These results suggested that CD55^+^ cells were more likely to be earlier‐stage granulosa cells than AMH‐EGFP^+^ cells. We have tried to culture the early‐stage CD55^+^ hiGC to test whether they can develop into the late‐stage hiGC. However, we did not observe the development of AMH‐EGFP^+^ cells from CD55^+^ cells. We think it is because the cells generated by forced lineage change are terminally differentiated cells which cannot undergo further proliferation and differentiation.

### 3.6 The effects of hiGC on oocyte maturation

**FIGURE 6:**
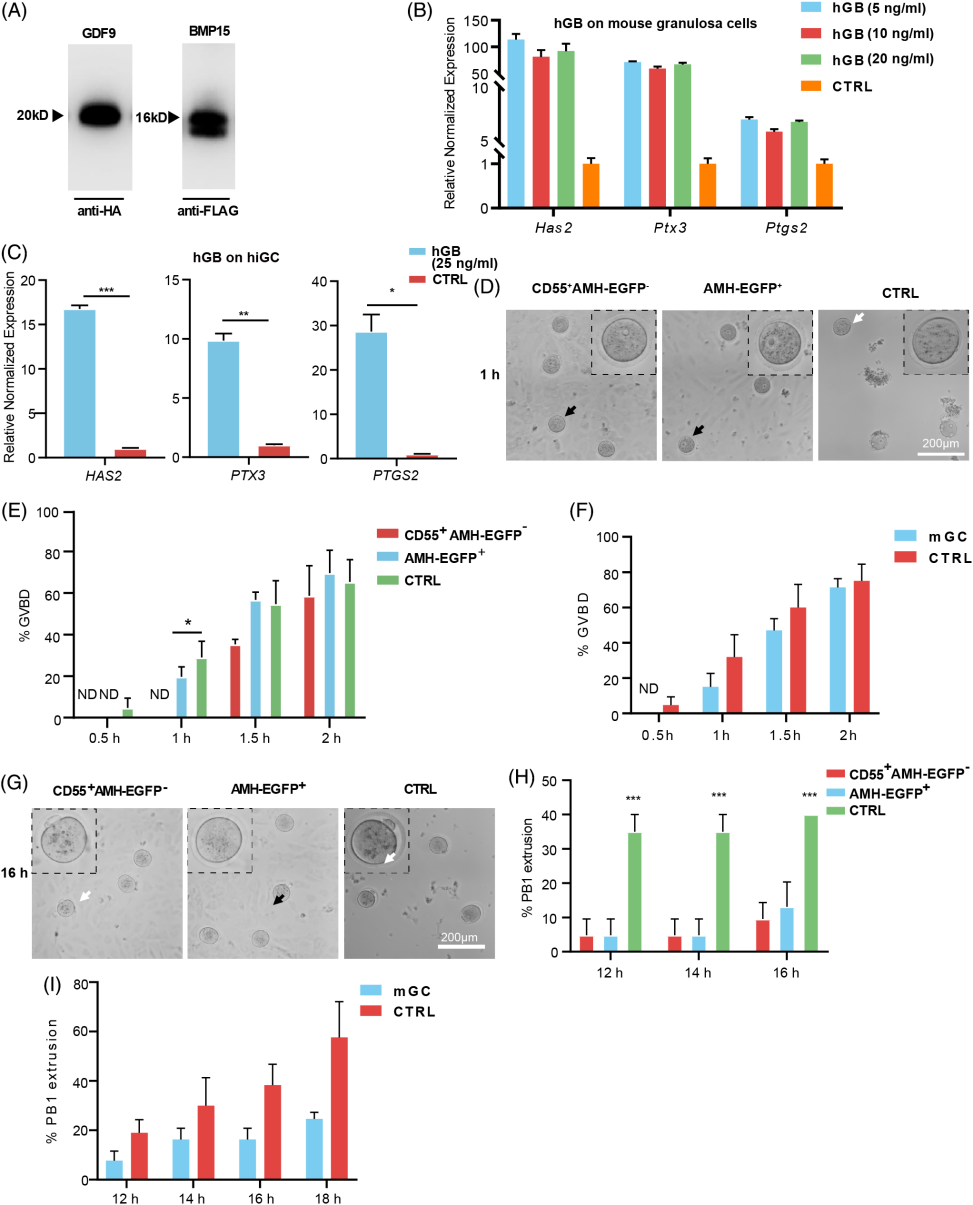
The interactions between hiGC and oocyte. (A) GDF9 and BMP15 were detected with western blot in anti‐FLAG agarose purified proteins. (B) The expression of cumulus expansion related genes. Expression were normalized to *GAPDH*. (C) The expression of cumulus expansion related genes in hGB treated hiGC. Expression we normalized to *GAPDH*. (D) Representative photographs of GV, GVBD oocytes in AMH‐EGFP^+^, CD55^+^AMH‐EGFP^−^ and CTRL group. Black arrows indicate GV stage oocytes and white arrows indicate GVBD oocytes. A magnified view of the oocyte designated by arrow was provided on the right corner. Scale bar = 200 μm. (E) Percentages of GVBD in AMH‐EGFP^+^, CD55^+^AMH‐EGFP^−^ and CTRL group at different timepoints. (*n* = 21 per group) (F) Percentages of GVBD in mouse granulosa cells (mGC) co‐cultured group and CTRL group at different timepoints. (*n* = 21 per group). (G) Representative photographs of first body extrusion (PB1) in oocytes of AMH‐EGFP^+^, CD55^+^AMH‐EGFP^−^ and CTRL group. White arrow and black arrow indicate oocyte with and without PB1 respectively. A magnified view of the oocyte designated by arrow was provided on the left corner. Scale bar = 200 μm. (H) Percentages of PB1 extrusion in AMH‐EGFP^+^, CD55^+^AMH‐EGFP^−^ and CTRL group at different timepoints (*n* = 21 per group). (I) Percentages of PB1 extrusion in mGC co‐cultured group and CTRL group at different timepoints (*n* = 21 per group). Data were means ± SEM of three independent experiments. For C, *p* values were determined by two‐tailed Student's *t*‐test. For D and G, *p* values were determined by one‐way ANOVA. *p < 0.05, ***p < 0.01, ***p < 0.001. ND, not detected.

GDF9 and BMP15 are key oocyte secreted factors that regulate the differentiation and proliferation of granulosa cells.^1^, ^31^, ^32^ We constructed a vector containing both N‐terminal HA‐tagged GDF9 and FLAG‐tagged BMP15 and purified the heterodimer as previously described^3^ (Figure S6). Purified hGDF9:BMP15 heterodimer were detected (Figure 6A) and exhibited stimulating effect on mouse granulosa‐cell markers^3^ (Figure 6B). Similarly, the purified heterodimers stimulated the expression of *HAS2*, *PTX3* and *PTGS2* in AMH‐EGFP^+^ hiGC cells (Figure 6C), indicating that AMH‐EGFP^+^ cells responded to oocyte secreted hGDF9:BMP15 heterodimer as previously reported in cumulus granulosa cells.

Previous study reported that granulosa cells of preovulatory follicle maintain oocyte in meiotic arrest to prevent the precocious maturation of oocyte.^33^, ^34^ To test the effect of the hiGC on oocyte meiotic resumption, AMH‐EGFP^+^ or CD55^+^AMH‐EGFP^−^ hiGC were co‐cultured with mouse oocytes of germ vesicle (GV) stage. The germ vesicle breaking down (GVBD) is a signature event occurs after meiosis resumes in oocytes. Through live cell imaging, occurrence of GVBD was observed to be delayed in AMH‐EGFP^+^ or CD55^+^AMH‐EGFP^−^ hiGC co‐culture group compared to the control group without co‐culture (Figure 6D,E). At 1 h, the percentage of GVBD was significantly lower in the AMH‐EGFP^+^ group (19%) and the CD55^+^AMH‐EGFP^−^ group (0%) than in the control group (29%). However, there was no significant difference of GVBD percentage between the two co‐culture groups and control group after 2 h. These results suggested that co‐culture with AMH‐EGFP^+^ or CD55^+^AMH‐EGFP^−^ hiGC delayed GVBD but did not affect the percentage of final GVBD. Consistently, mouse granulosa cells also exhibited similar effect of delaying GVBD of the oocytes (Figure 6F).

First polar body (PB1) extrusion indicates progression of meiosis in oocytes to meiosis II. Through live cell imaging, it was found that the percentage of PB1 extrusion of oocytes co‐cultured with AMH‐EGFP^+^ or CD55^+^AMH‐EGFP^−^ hiGC was significantly lower at 12, 14 and 16 h observation time points (Figure 6G,H), showing inhibitory effect of AMH‐EGFP^+^ and CD55^+^AMH‐EGFP^−^ hiGC on PB1 extrusion. Mouse granulosa cells showed similar inhibitory effect on PB1 extrusion (Figure 6I). These results suggested that both AMH‐EGFP^+^ and CD55^+^AMH‐EGFP^−^ hiGC delayed meiotic resumption of GV stage oocytes.

### 3.7 CD55 ^+^ hiGC alleviate DHEA‐induced PCOS

**FIGURE 7:**
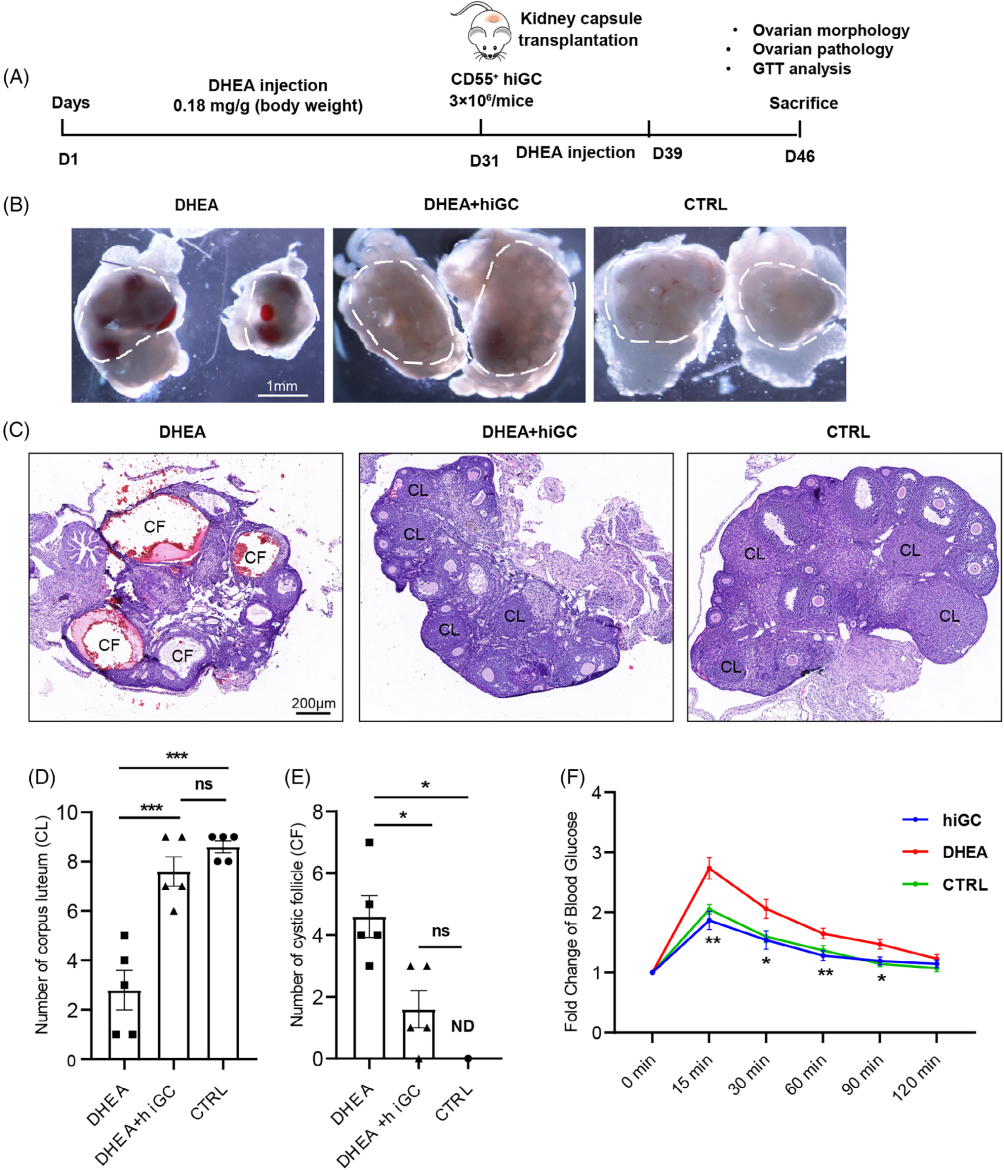
CD55 ^+^ hiGC alleviate DHEA‐induced PCOS. (A) Scheme of the experimental design. (B) Morphology of ovaries at day 46. Scale bar = 1 mm. (C) Haematoxylin and eosin staining of representative ovaries. Scale bar = 200 μm. (D) Quantitative analysis of corpus lutea (CL). (*n* = 5 mice per group). (E) Quantitative analysis of cystic follicles (CF) (*n* = 5 mice per group). (F) GTT analysis (*n* = 9 in control group, *n* = 5 in DHEA and DHEA+hiGC group). *p* values were determined by one‐way ANOVA or two‐way ANOVA. *p < 0.05, **p < 0.01, ***p < 0.001; ns, no statistical significance; ND, not detected.

PCOS is a major reproductive and endocrine disorder in women. The accumulation of excessive testosterone plays an important role in the development of PCOS. Currently, the therapy for PCOS mainly relies on chemically synthesized estradiol and progesterone.^8^ However, human‐derived natural steroid therapy and the conversion of excessive testosterone have not been reported. The ability of hiGC to secret human‐derived natural steroid and convert testosterone to estradiol suggested that transplanting hiGC to PCOS individuals may alleviate PCOS syndrome. Although there is study that suggested cell transplantation as a therapeutic strategy for PCOS,^35^ we did not find pre‐existing studies that directly use granulosa cells as transplants for PCOS treatment. Therefore, we established a DHEA‐induced PCOS mouse model as previously described^18^ and tested the effect of hiGC transplantation on the polycystic ovaries (Figure 7A).

The PCOS mice (DHEA) showed serious damage with ovarian haemorrhage (Figure 7B), significantly decreased number of corpus lutea (CL) and increased number of cystic follicles (CF) in their ovaries, as well as insulin resistance (Figure 7C–F). Mice that received CD55^+^ hiGC transplantation (DHEA+hiGC) showed significant recovery without serious ovarian haemorrhage (Figure 7B). Notably, transplantation of CD55^+^ hiGC significantly increased the number of corpus lutea and decreased the number of cystic follicles (Figure 7C–E) compared with the DHEA group. GTT analysis demonstrated that mice that received CD55^+^ hiGC transplantation showed significant rescue of insulin resistance (Figure 7F). Furthermore, we have tried the transplantation of AMH‐EGFP^+^ hiGC to PCOS mice and observed obvious alleviation of ovary damage from histological analysis (Figure S7A). Although preliminary experiments showed that AMH‐EGFP^+^ hiGC had a relatively good therapeutic effect, the proportion of AMH‐EGFP^+^ hiGC is much lower than that of CD55^+^ hiGC. This makes it challenging to obtain a sufficient quantity of cells for future clinical trials. Therefore, we have opted to proceed with further animal experiments using CD55^+^ hiGC. We then tested whether the transplantation of hiGC affected the serum levels of testosterone and estradiol in PCOS mice. An oral‐contraceptive group (DHEA+OC) was introduced as an positive control to mimic the clinical treatment of PCOS. ELISA results showed that the DHEA group exhibited the highest testosterone level (significantly higher than the control (*p* < 0.05) and lowest estradiol level (Figure S7B,C). The DHEA+OC group showed significantly elevated estradiol level (*p* < 0.05) compared with the DHEA group, while the testosterone level was only mildly decreased and still significantly higher than control (*p* < 0.01) (Figure S7B,C). In comparison, hiGC transplantation restored the levels of both hormones, showing no significant difference from the control group (Figure S7B,C). These results suggested that transplantation of hiGC alleviated ovarian damage and insulin resistance, and was able to adjust the hormone concentration to relatively normal levels in the DHEA‐induced PCOS mice.

## 4 CONCLUSIONS

In this study, we established a reprogramming system in which FOXL2 and NR5A1 transcription factors combination induced human fibroblasts into two kinds of granulosa‐like cells with distinct transcriptomes and steroidogenic activities. AMH‐EGFP^+^ cells were similar to human cumulus granulosa cells in transcriptome profile and produced high levels of estradiol and progesterone. In contrast, CD55^+^ cells produced high level of estradiol but low level of progesterone. Transcriptome analysis confirmed that CD55^+^AMH‐EGFP^−^ cells expressed higher levels of early‐stage granulosa cell‐related genes and lower levels of late‐stage granulosa cell‐related genes. Hence, through the overexpression of FOXL2 and NR5A1, we reprogrammed and isolated two populations of granulosa cells resembling early‐ and late‐stage granulosa cells. Notably, NR5A1 is a transcriptional factor not only expressed in granulosa cells but also in theca cells and Leydig cells. However, theca cells do not express AMH and high levels of FOXL2, and the ability to secret oestrogen is significantly lower than granulosa cells.^36^ Moreover, Leydig cells are male‐specific cells that do not express FOXL2.^37^ We believe that our cells induced by overexpression of FOXL2 and NR5A1 cannot be theca cells or Leydig cells. A recent study reported the differentiation of human‐induced pluripotent stem cells into granulosa‐like cells through the overexpression of NR5A1 and either RUNX1 or RUNX1. This is consistent with our finding that NR5A1 overexpression participates in the induction of granulosa‐like cells. However, unlike our research, which isolated early‐ and late‐stage granulosa‐like cells using different markers, the granulosa‐like cells in that study were isolated using FOXL2, which is expressed in nearly all stages of granulosa cells. Thus, different stages of granulosa‐like cells were not divided in that research, which is important because different stages of granulosa cells differ greatly and support different stages of germ cells.^38^

Currently, estradiol and progesterone used in clinical practice are mainly derived from chemical synthesis. Estradiol or progesterone produced by chemical synthesis may contain isomers that are not natural and may result in biological activities that differ from steroid hormones produced in humans. hiGC secreted high levels of oestrogen or progesterone, which can be an ideal system to produce human‐derived natural steroid hormones. Exploiting the steroidogenic activity of secreting human‐derived natural steroid and converting testosterone to estradiol, we validated that CD55^+^ hiGC transplantation into PCOS mouse model alleviated the PCOS syndromes. Recent studies have reported that bone marrow mesenchymal stem cell transplantation for the alleviation of PCOS in mice lacks the conversion of extra testosterone, which is the leading cause of PCOS.^39^, ^40^ Our strategy of directly converting extra testosterone to estradiol might restore steroidogenic homeostasis in the PCOS ovary and ameliorate syndromes, suggesting that hiGC transplantation can be developed as a novel cellular therapy for PCOS patients.

As hiGC includes two populations of granulosa cells resembling early‐ and late‐stage granulosa cells, they can be used as a model to study the developmental programs and function of human granulosa cells, as well as the mechanisms of female reproductive disease caused by granulosa cells deficiencies. FOXL2 and NR5A1 are reported to play pivotal roles in the development of mouse granulosa cells,^11^, ^23^ but their contribution to the development of human granulosa cells remains unclear. Interestingly, *FOXL2* and *NR5A1* mutations were previously reported to be closely associated with POI.^9^ The hiGC reprogramming system induced by FOXL2 and NR5A1 overexpression provides an in vitro model to further study the underlying mechanisms of POI caused by *FOXL2* and *NR5A1* mutations or other putative mutation in human granulosa cells. Moreover, the hiGC reprogramming system may provide a platform to identify small molecules or drugs for treating granulosa cell‐related diseases.

**FIGURE 8:**
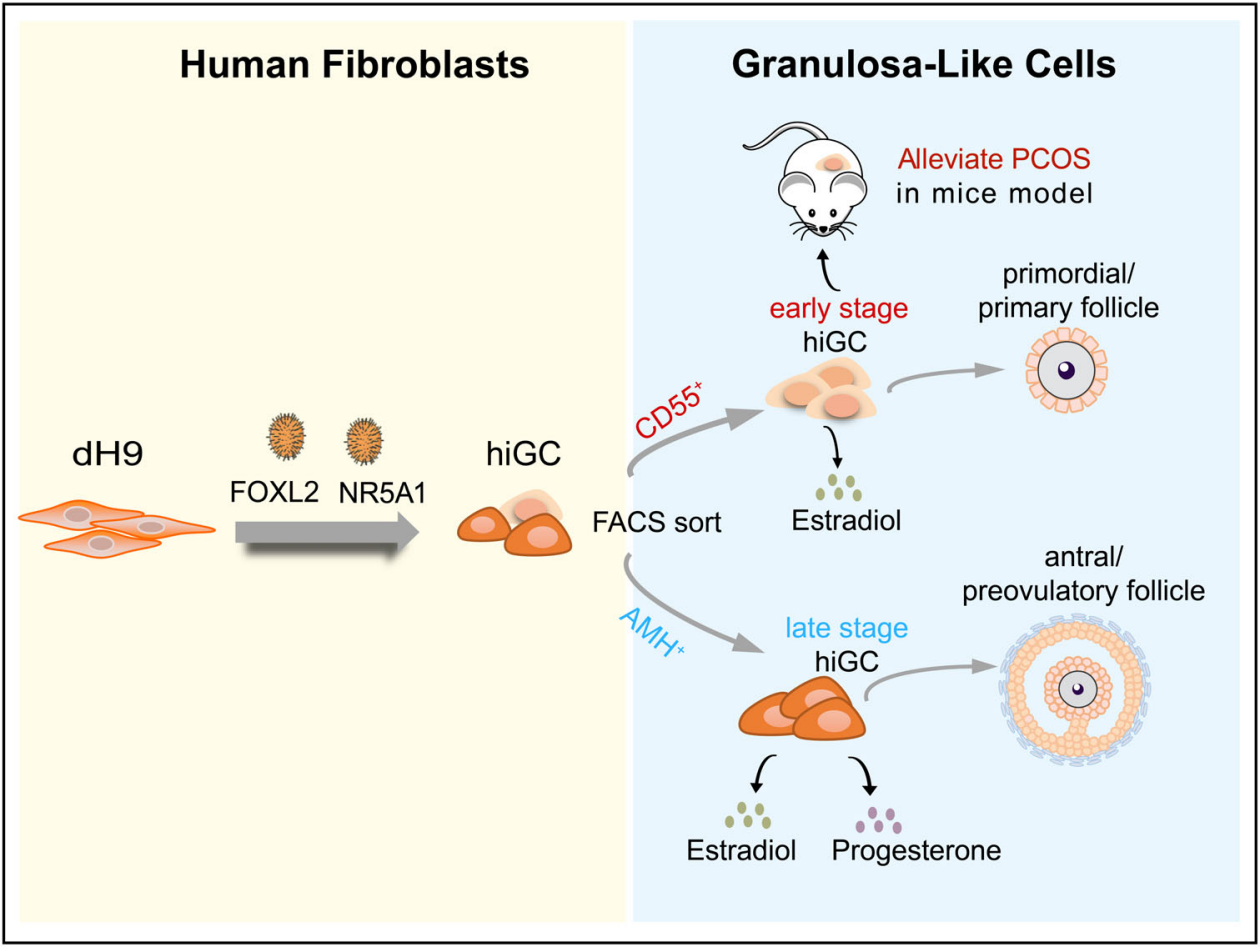
The model of in vitro reprogramming of human fibroblasts into hiGC. Overexpression of FOXL2 and NR5A1 reprogrammed dH9 fibroblast cells into AMH‐EGFP^+^ hiGC and CD55^+^ hiGC similar to late‐stage and early‐stage granulosa cells respectively. AMH‐EGFP^+^ hiGC secret high levels of estradiol and progesterone, and CD55^+^ hiGC secret high level of estradiol. CD55^+^ hiGC transplantation can alleviate DHEA‐induced PCOS and has potential for cell therapy of PCOS.

In summary, this study presented direct reprogramming of human granulosa‐like cells using two transcriptional factors, FOXL2 and NR5A1. The reprogrammed hiGC can be divided into early‐ and late‐stage populations with different steroidogenic activities. Transplantation of CD55^+^ hiGC alleviated PCOS syndromes in the DHEA‐induced PCOS mice (Figure 8). These hiGC provide a novel platform for basic research and clinical therapy.

## AUTHOR CONTRIBUTIONS

*Conceptualization*: Kehkooi Kee, Fan Wen. *Methodology*: Kehkooi Kee, Fan Wen. *Investigation*: Fan Wen, Yuxi Ding, Jing Du, Shen Zhang, Mingming Wang. *Visualization*: Fan Wen, Mingming Wang, Yuxi Ding. *Writing—original draft*: Fan Wen. *Writing—review & editing*: Kehkooi Kee, Fan Wen, Yuxi Ding. *Supervision*: Kehkooi Kee.

## FUNDING INFORMATION

This work was supported by the National Natural Science Foundation of China (82071597); the Ministry of Science and Technology of China (2022YFA0806301, 2021YFA0719301); Tsinghua‐Peking Center for Life Sciences.

## CONFLICT OF INTEREST STATEMENT

The authors have no conflict of interest to disclose.

## INFORMED CONSENT STATEMENT

Informed consent was obtained from all individual participants included in the study. Patients signed informed consent regarding publishing their data.

## Supporting information


**Data S1:** Supporting Information.

## Data Availability

The datasets generated during and/or analysed during the current study are available in the GEO repository (the accession number is GSE203070). Any additional information required to reanalyse the data reported in this paper is available from the corresponding author upon request.
