# Supplementary material for: FOXL2 and NR5A1 induce human fibroblasts into steroidogenic ovarian granulosa‐like cells
Source: Cell Prolif. 2024 Jan 8;57(5):e13589. doi: 10.1111/cpr.13589 (PMC11056703; doi:10.1111/cpr.13589)
Supplement: Supplementary file 1 — Data S1: Supporting Information. [file CPR-57-e13589-s001.docx]

# Supporting information

# Title

# FOXL2 and NR5A1 Induce Human Fibroblasts into Steroidogenic Ovarian Granulosa-Like Cells

Fan Wen^1#^, Yuxi Ding^1#^, Mingming Wang^1^, Jing Du^1^, Shen Zhang^2,3^, Kehkooi Kee^1^*

# Affiliations

^1^ The State Key Laboratory for Complex, Severe, and Rare Diseases; SXMU-Tsinghua Collaborative Innovation Center for Frontier Medicine; Department of Basic Medical Sciences, School of Medicine, Tsinghua University, Beijing, 100084, China.

^2^ Reproductive Medicine Center, The First Affiliated Hospital, Wenzhou Medical University, Wenzhou 325000, China.

^3^ Reproductive Medicine Center, Department of Obstetrics and Gynecology, The Second Affiliated Hospital, Chongqing Medical University, Chongqing 400010, China.

^#^ These authors contributed equaaly to this study

* Corresponding author, Kehkooi Kee, Email: [kkee@tsinghua.edu.cn](mailto:kkee@tsinghua.edu.cn)


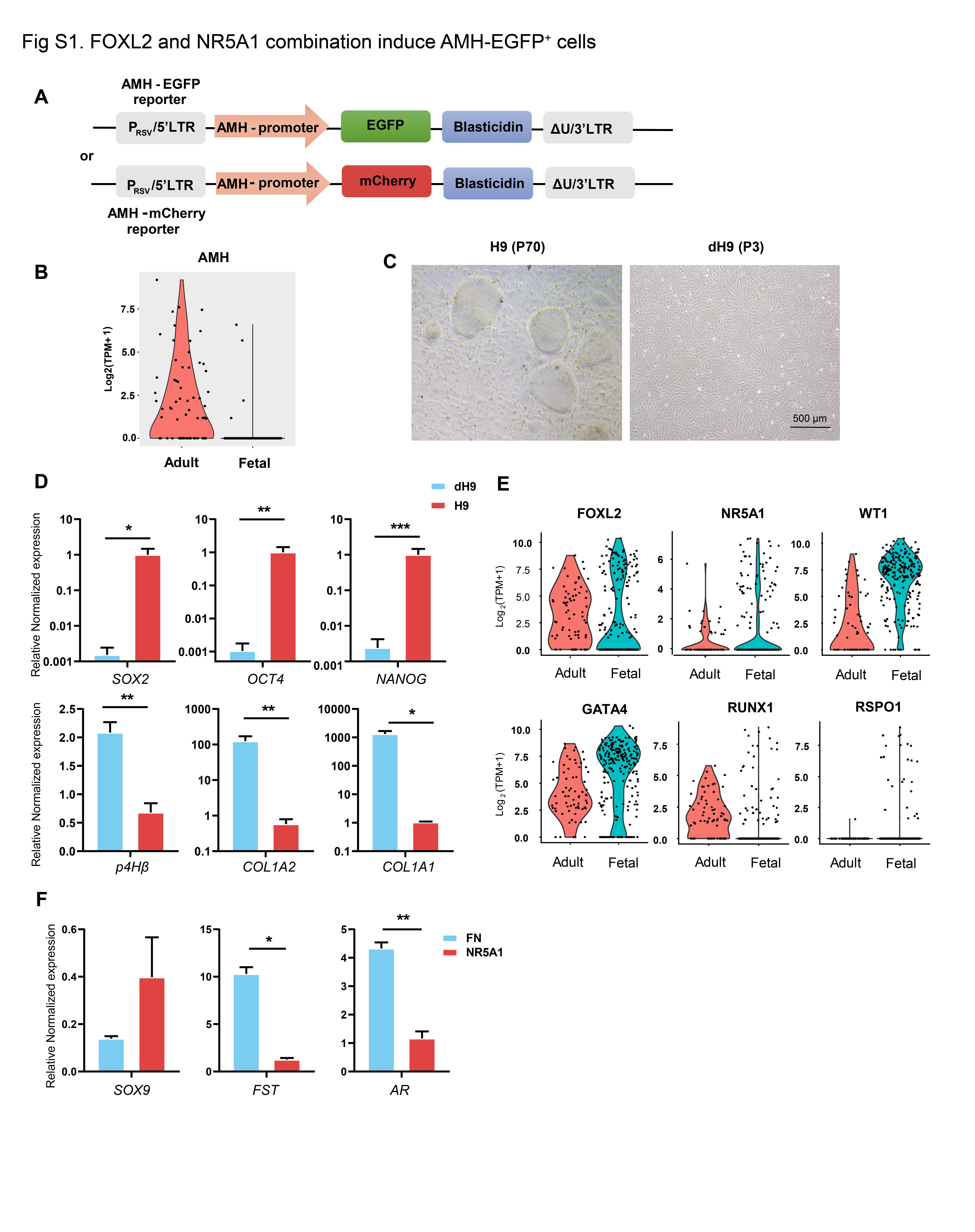


## Figure S1. FOXL2 and NR5A1 combination induce AMH-EGFP^+^ cells

(A) Scheme of vectors of AMH-EGFP reporter and AMH-mCherry reporter. (B) The expression of AMH in human fetal and adult granulosa cells. Single-cell RNA-sequencing data were downloaded from Li et al., 2017(Li et al., 2017) and Zhang et al., 2018(Zhang et al., 2018) (C) The morphology of H9 hESC and passage3 (P3) dH9 cells. (D) the expression of indicated genes in H9 and dH9 were measured by qRT-PCR. Expression were normalized to *GAPDH.* (E) The expression of six candidate factors in human fetal and adult granulosa cells. Single-cell RNA-sequencing data were downloaded from Li et al., 2017(Li et al., 2017) and Zhang et al., 2018(Zhang et al., 2018). (F) The expression of *SOX9, FST, AR* in AMH-EGFP^+^ cells of FN or NR5A1 group were measured by qRT-PCR (n = 3). Expression were normalized to *GAPDH*.

**
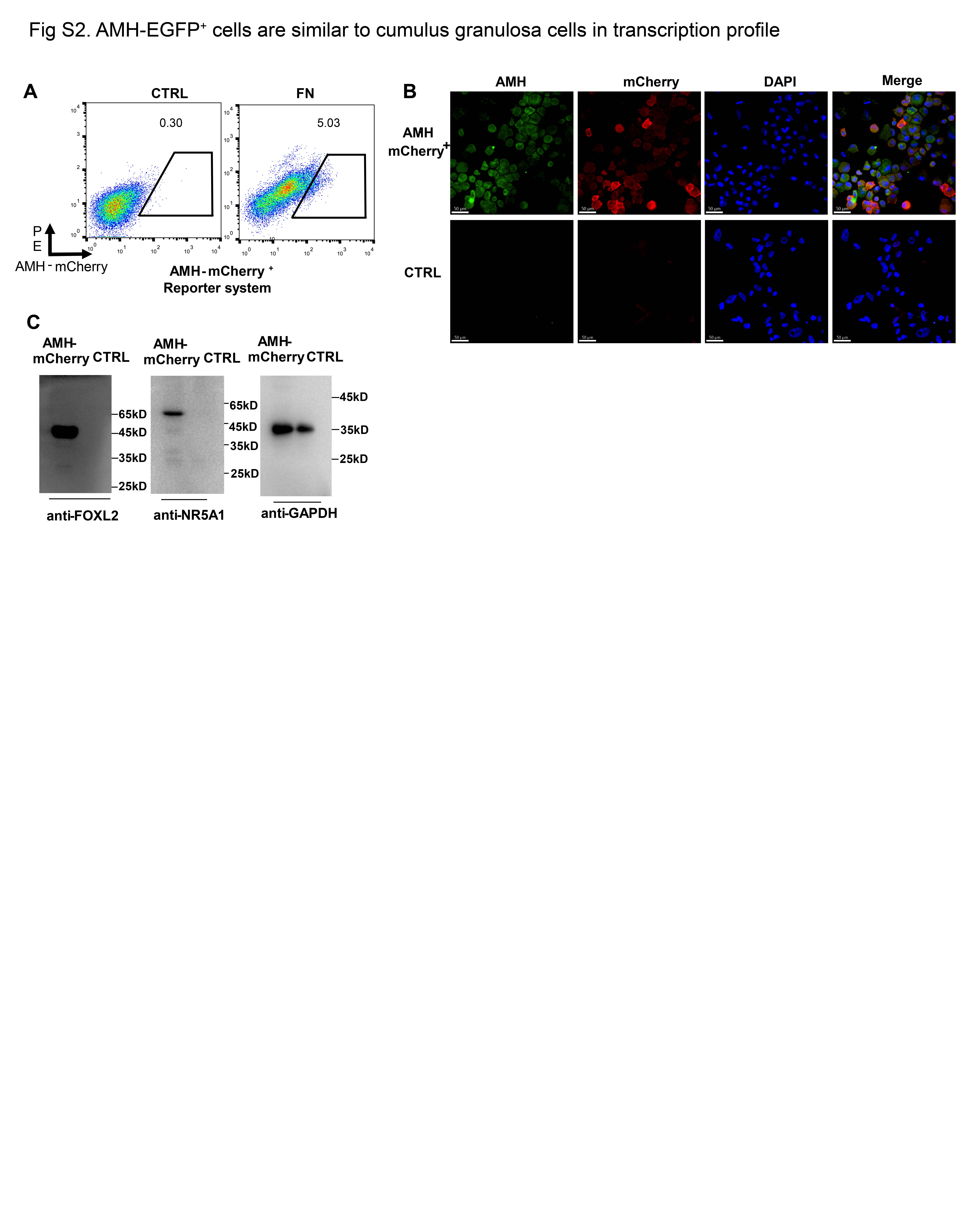
**

**Figure S2. AMH-EGFP^+^ cells are similar to cumulus granulosa cells in transcription profile**

(A) Flow cytometric analysis of AMH-mCherry^+^ cells in AMH-mCherry reporter system. CTRL was transduced with p2k7 empty virus. (B) Immunofluorescence staining of AMH and mCherry in day11 sorted AMH-mCherry^+^ cells. Scale bar = 50 μm. (C) The expression of FOXL2 and NR5A1 were detected in day11 sorted AMH-mCherry^+^ cells by Western blot.


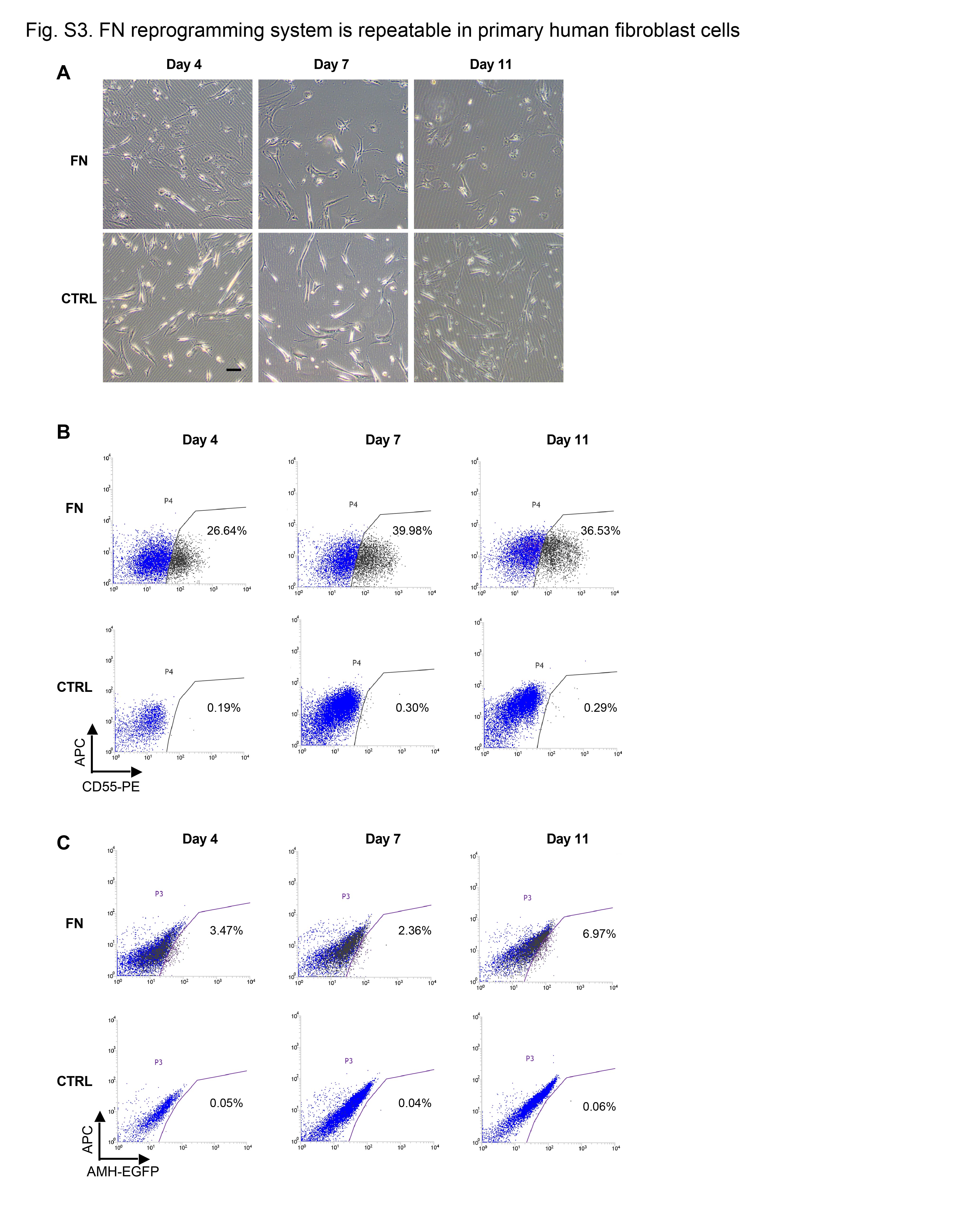


**Figure S3. FN reprogramming system is repeatable in primary human fibroblast cells**

(A) The morphology of FN-overexpressed WI38 at day 4, 7 and 11. Scale bar = 100 μm. (B) Flow cytometric analysis of CD55^+^ cells at day 4, 7 and 11. (C) Flow cytometric analysis of AMH-EGFP^+^ cells at day 4, 7 and 11.


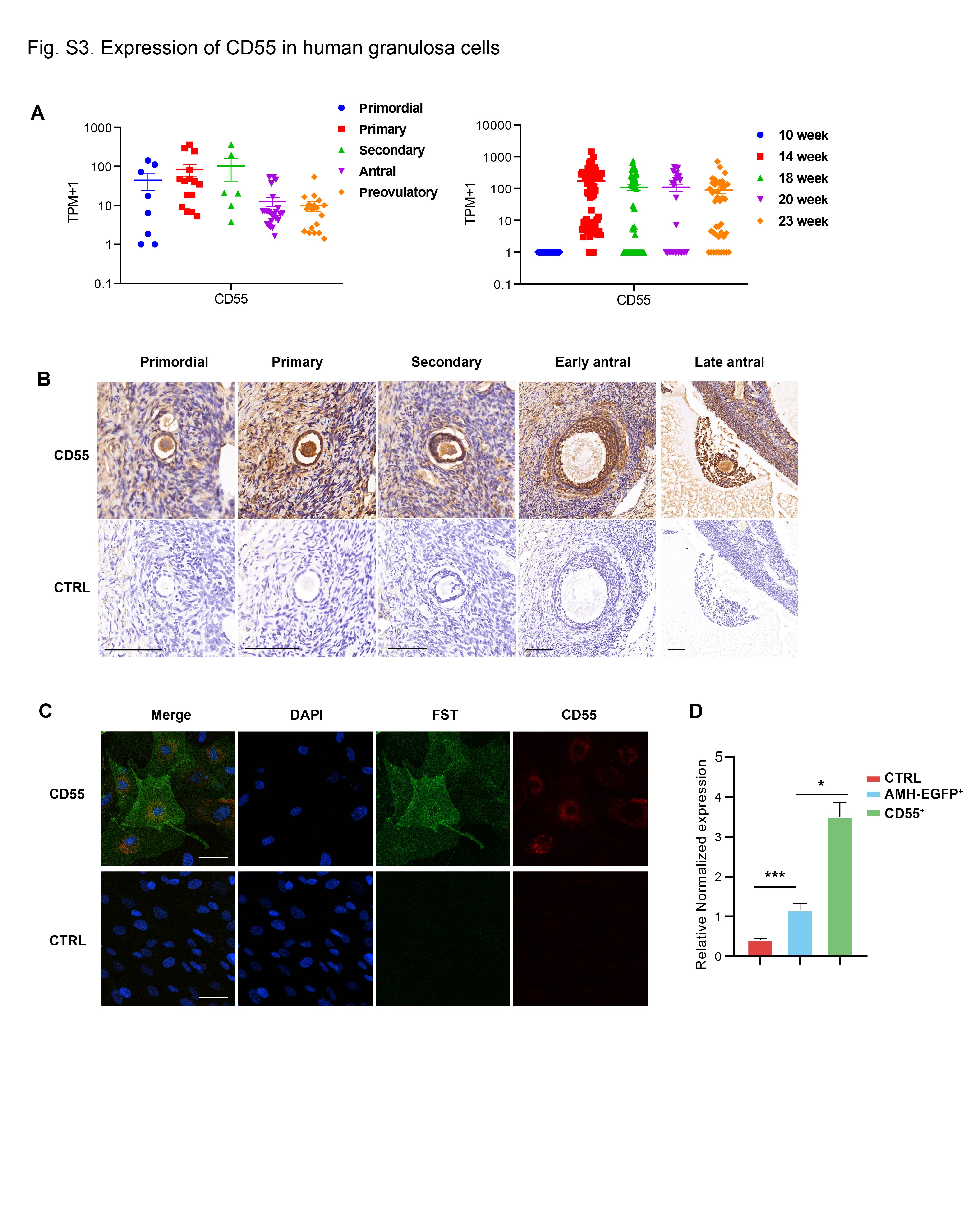


## Figure S4. Expression of CD55 in human granulosa cells

(A) Expression of CD55 in human gonadal granulosa cells and adult granulosa cells of different stage follicle. Single-cell RNA-sequencing data were downloaded from Li et al., 2017 (Li et al., 2017) and Zhang et al., 2018 (Zhang et al., 2018). (B) immunohistochemistry staining of CD55 in human ovarian paraffin section. Scale bar = 100 μm. (C) Immunofluorescence staining of FST in CD55^+^ cells. (D) the expression of *FST* was measured by qRT-PCR.


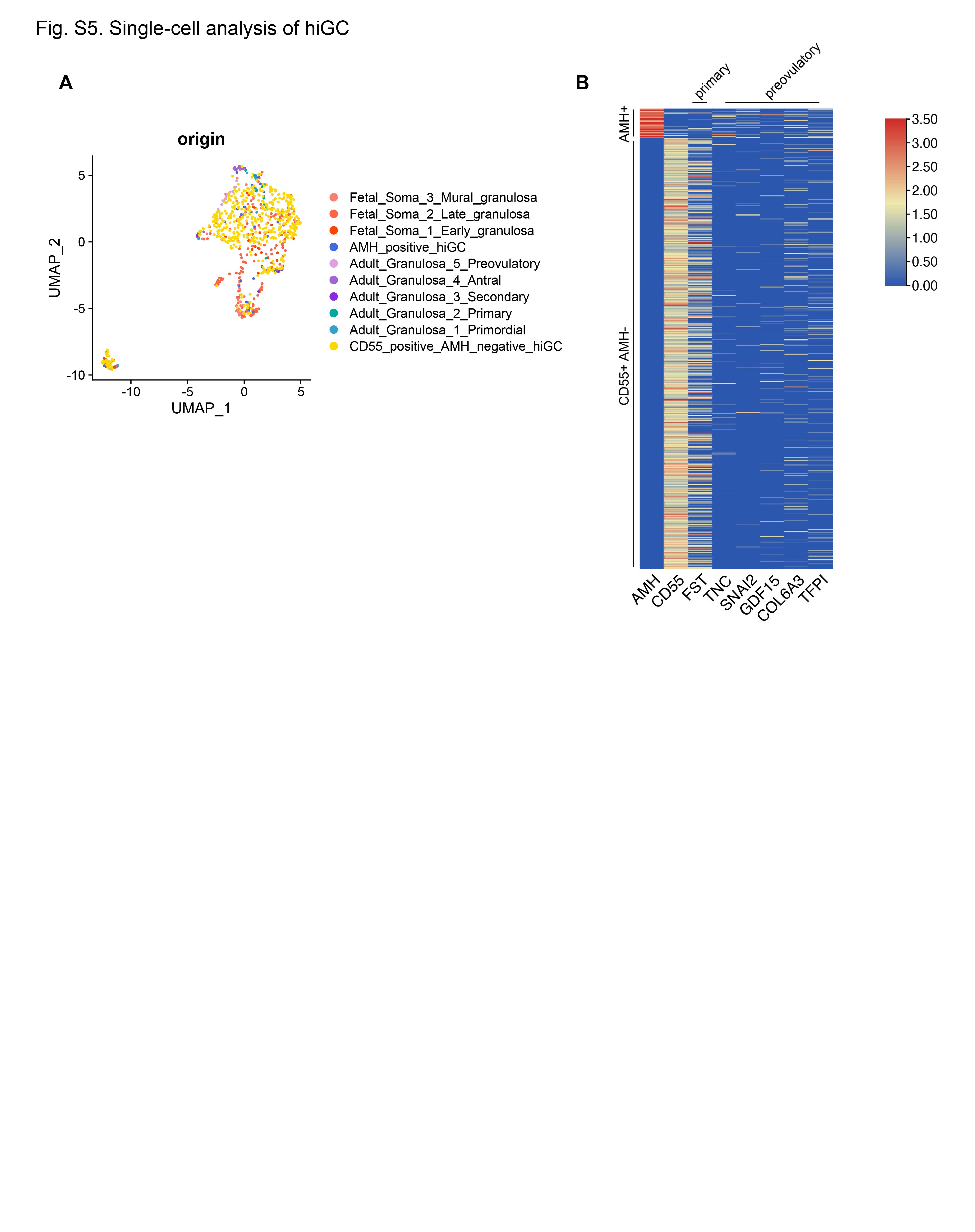


## Figure S5. Single-cell analysis of hiGC

(A) UMAP plot of the integrated dataset of AMH-EGFP^+^ hiGC, CD55^+^AMH-EGFP^-^ hiGC, and in-vivo granulosa cells. (B) Expression of granulosa markers in AMH-EGFP^+^ and CD55^+^AMH-EGFP^-^ cells.


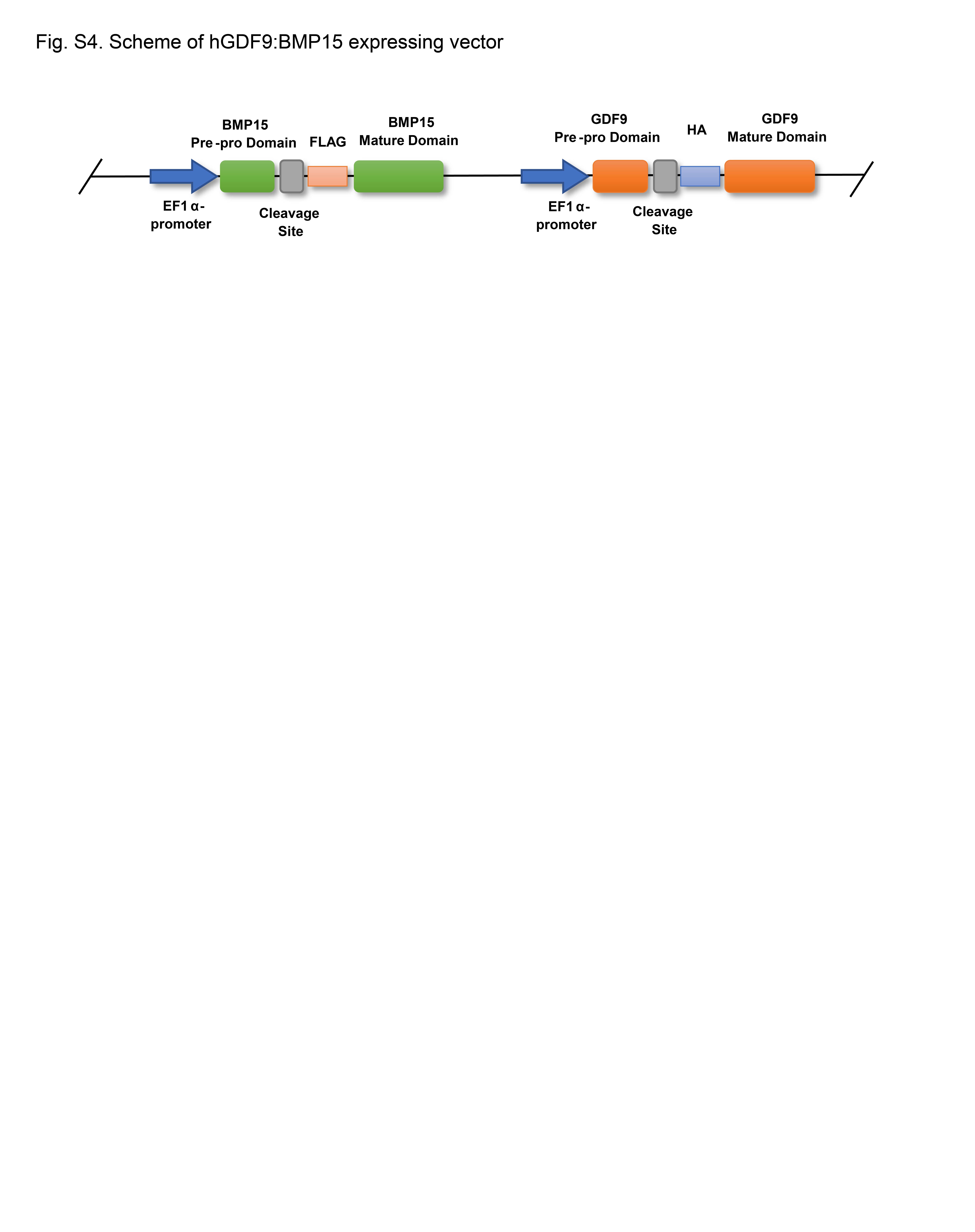


## Figure S6. Scheme of hGDF9:BMP15 expressing vector

N-terminal of GDF9 and BMP15 is HA tagged and FLAG tagged respectively.


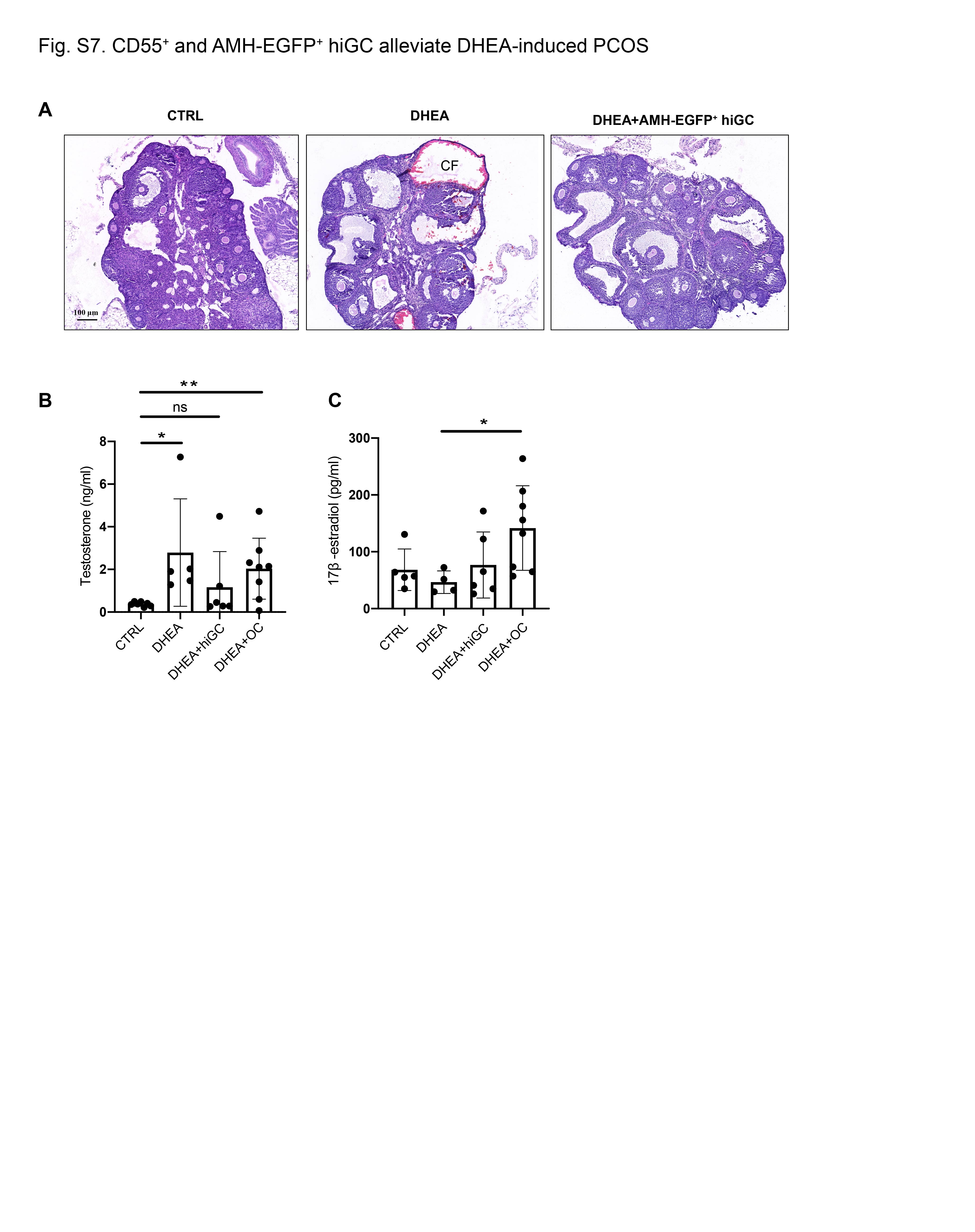


## Figure S7. CD55^+^ and AMH-EGFP^+^ hiGC alleviate DHEA-induced PCOS

(A) Hematoxylin and eosin staining of representative ovaries (n=3 mice per group). CF: cystic follicles. Scale bar = 100 μm. (B) ELISA measurement of serum level of testosterone (n=5 in control and DHEA group, n=6 DHEA+hiGC group, n=8 in DHEA+OC group). (C) ELISA measurement of serum level of 17β-estradiol (n=5 in control group, n=4 in DHEA group, n=6 DHEA+hiGC group, n=8 in DHEA+OC group). p values were determined by one-way ANOVA. * p < 0.05, ** p <0.01, ns, no statistical significance.
